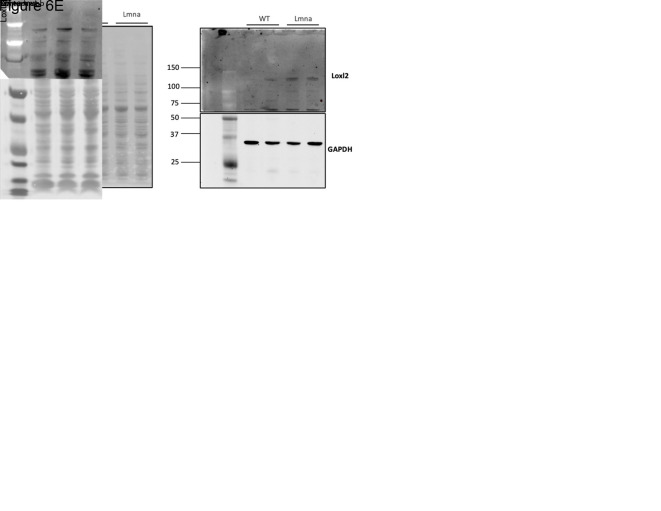# Simtuzumab Attenuates Loxl2-Mediated Extracellular Matrix Remodeling and Preserves Cardiac Function in *LMNA* Mutation-Induced Dilated Cardiomyopathy

**DOI:** 10.1161/CIRCHEARTFAILURE.125.013806

**Published:** 2026-03-17

**Authors:** Marie Kervella, Charlotta S. Behrens, Cécile Peccate, Zoheir Guesmia, Fiorella Grandi, Nathalie Mougenot, Anne Forand, Azzouz Charrabi, Guy Brochier, Ramaroson Andriantsitohaina, Sonia R. Singh, Thomas Eschenhagen, Albano C. Meli, Antoine Muchir

**Affiliations:** 1 Institute of Myology, Center of Research in Myology, Sorbonne University, Inserm, Paris, France (M.K., C.P., Z.G., F.G., A.F., A.M.).; 2 PhyMedExp, University of Montpellier, Inserm, CNRS, France (M.K., A.C., R.A., A.C.M.).; 3 Institute of Experimental Pharmacology and Toxicology, University Medical Center Hamburg-Eppendorf, Germany (C.S.B., S.R.S., T.E.).; 4 German Centre for Cardiovascular Research (DZHK), partner site North (C.S.B., S.R.S., T.E.).; 5 Phénotypage du petit animal, Inserm UMS28, Sorbonne University-UPMC Paris 06, Inserm, Paris, France (N.M.).; 6 Inovarion, F-75005 Paris, France (A.F.).; 7 Functional Unit of Neuromuscular Pathology, Neuropathology Department, Institute of Myology, CHU Pitié-Salpêtrière, Paris, France (G.B.).

**Keywords:** chromosomes, fibrosis, heart failure, lamins, point mutation

## Abstract

**BACKGROUND:**

Dilated cardiomyopathy caused by *LMNA* mutations is a severe cardiac condition marked by arrhythmias, contractile dysfunction, and excessive myocardial fibrosis, which collectively impair left ventricular function and increase the risk of heart failure. Although the disease has been well characterized, a lack of insight into the pathogenesis has impeded the development of therapies.

**METHODS:**

Here, we employed human induced pluripotent stem cells (hiPSCs) derived from a patient carrying a *LMNA* point mutation (c.665A>C, p.His222Pro), alongside a murine model carrying the same mutation, to investigate the functional and molecular abnormalities driving dilated cardiomyopathy.

**RESULTS:**

We demonstrated that *LMNA* patient-derived cardiomyocytes and engineered heart tissues exhibited elevated diastolic calcium levels and reduced sensitivity to external calcium, respectively, as well as hypocontractility. These cells also displayed nuclear shape abnormalities in 2-dimensional and 3-dimensional, a hallmark of *LMNA*-associated dilated cardiomyopathy, associated with disrupted chromosome spatial organization and altered gene expression profiles. Transcriptomic analysis revealed dysregulation of extracellular matrix remodeling and significant upregulation of Loxl2 in mutated hiPSC–cardiomyocytes, hiPSC–engineered heart tissues, and mice. Treatment with Simtuzumab, a Loxl2 inhibitor, effectively prevented cardiac dysfunction and fibrosis in vivo.

**CONCLUSIONS:**

Taken together, our findings underscore the crucial role of Loxl2 as a therapeutic target and suggest that its inhibition could be a promising strategy to preserve cardiac function in *LMNA*-associated dilated cardiomyopathy.

**WHAT IS NEW?**

- The *LMNA* p.H222P mutation triggers chromosome repositioning associated with the dysregulation of extracellular matrix-related genes in cardiac muscle cells.
- Loxl2 gene expression is commonly upregulated in cardiac muscle cells expressing mutated A-type lamins.

**WHAT ARE THE CLINICAL IMPLICATIONS?**

- *LMNA*-associated dilated cardiomyopathy is characterized by excessive myocardial fibrosis.
- The inhibition of Loxl2 prevented cardiac fibrosis.
- The inhibition of Loxl2 halted the progression of the disease.

Inherited cardiomyopathies represent a major cause of heart disease, often manifesting during adolescence or early adulthood. Among these, dilated cardiomyopathy (DCM) is characterized by left ventricular dilation, impaired systolic function, and myocardial fibrosis.^1^ To date, mutations in over 40 genes have been identified as contributing to the development of DC.^2^ Among them, mutations in the *LMNA* gene, which encodes the nuclear A-type lamins, are recognized as the second most common genetic cause of familial DCM—highlighting the importance of understanding their pathogenic mechanisms.^3^ *LMNA*-associated DCM is clinically distinct due to its rapid progression, early and widespread myocardial fibrosis, high arrhythmic burden, and accelerated onset of heart failure, compared with most other forms of inherited cardiomyopathy.^4–7^ Although the genetic origins of the disease are well established, the molecular and cellular mechanisms driving disease progression remain incompletely understood, posing a significant barrier to therapeutic development.^8^ *LMNA* mutations lead to structural abnormalities of the nuclear envelope, including disruption of nuclear integrity and altered nuclear morphology—a hallmark of *LMNA*-associated DCM.^9,10^ These nuclear defects are believed to impact key cellular processes and may contribute to tissue-specific manifestations of the disease, particularly in cardiac muscle. Elucidating how *LMNA* mutations give rise to heart-specific dysfunction will be crucial for uncovering the pathways that drive disease progression and for identifying novel therapeutic targets.

Nuclear A-type lamins are part of the nuclear lamina, a filamentous network on the inner nuclear membrane.^11^ The nuclear lamina is not only structurally important, but it also interacts with specific regions of genomic DNA called lamina-associated domains (LADs), which are chromatin regions that are physically tethered to the inner nuclear membrane.^12,13^ LADs are typically transcriptionally repressed, and their positioning within the nucleus significantly regulates gene expression.^14^ However, some LADs could be repositioned, moving either away from or toward the nuclear lamina in a cell-type-specific manner. This repositioning is critical for regulating gene expression, and any disruptions in this process can lead to altered transcriptional programs that contribute to *LMNA*-associated DCM.^15^ Despite growing recognition of the nuclear lamina’s importance, much remains unclear regarding how these interactions influence cellular function, how they mechanistically drive fibrosis, and ultimately contribute to *LMNA*-associated DCM.

In this study, we sought to explore the hypothesis that *LMNA* variants may disrupt chromatin organization, leading to Loxl2-mediated extracellular matrix (ECM) dysfunction, which may ultimately contribute to DCM. Through in vitro and in vivo functional, morphological, and transcriptomic high-throughput experiments, our findings revealed, for the first time, that *LMNA* variants cause distinct alterations in the chromosome spatial arrangement in induced pluripotent stem cells (hiPSCs) from a patient carrying a *LMNA* point mutation. Among the various *LMNA* mutations associated with DCM, the p.H222P point mutation—a missense mutation resulting in the substitution of histidine with proline at position 222 of the lamin A/C protein—has been extensively studied and is considered a representative model of *LMNA* missense mutations due to its consistent phenotypic presentation in both human patients and animal models.^16,17^ As such, p.H222P serves as a paradigm for studying how single amino acid substitutions in lamin A/C can drive tissue-specific disease and offers a robust platform for preclinical therapeutic exploration. These alterations in the chromosome spatial arrangement were associated with significant changes in the expression of *Loxl2*, a key mediator of ECM remodeling. We demonstrated that pharmacological inhibition of *Loxl2* using Simtuzumab effectively preserved cardiac function and mitigated cardiac fibrosis in *LMNA*-mutated mice. This study provides novel insights into the pathogenic mechanisms underlying the disease and highlights *Loxl2* as a promising therapeutic target for addressing pathological cardiac remodeling.

## Methods

All supporting data are available within this article and its Supplemental Material.

### Ethics Statement

The study adhered to the principles of the Declaration of Helsinki.^18^

### hiPSC Lines

We used the patient-specific *LMNA* p.H222P hiPSC line (*LMNA* H222P) and its isogenic control (*LMNA* corr.H222P) as previously published. The generation and characterization of these lines, including validation of pluripotency markers and genome editing for the isogenic control, were reported in detail.^18^ All hiPSC lines were cultured under feeder-free conditions on Matrigel-coated plates as previously reported.^19^ All lines were routinely tested and confirmed to be negative for mycoplasma contamination (MycoAlert, Lonza). To avoid the accumulation of genomic instability, hiPSC lines were used for differentiation for a maximum of 10 passages after recovery from frozen stocks and then discarded. This controlled passage window prevents instability and minimizes the risk of karyotypic abnormalities; consequently, routine serial karyotyping over time was not required.

### Generation of Isogenic hiPSC Line LMNA corr.H222P

To generate the *LMNA* p.H222P isogenic control (corr.H222P), the *LMNA* H222P patient cell line was expanded in FTDA media and grown until 80% to 90% confluency.^20^ hiPSCs were dissociated with Accutase into single cells and nucleofected with Amaxa Solution P3 and 4 μL of ssODN (4 µmol/L; 5’-tctgtgtccttcctccaacccttccagGAGCTGCGTGAGACCAAGCGACGTCATGAGACCCGACTGGTGGAGATTGACAATGGGAAGCAGCGTGAGTTTGAGAGCCGGCTGGC -3’), and 1 μL RNP complex, Cas9 3NLS/sgRNA complex (5’- CCACCAGTCGGGTCTCAGGA -3’) following the manufacturer’s instructions (program CA137) for the 4D-NucleofectorTM (Lonza). For genomic analysis of the clones, regions of interest were amplified via polymerase chain reaction to assess editing efficiency and exclude the 10 most probable off-targets according to http://crispor.tefor.net/. Primer sequences: off-target (OT) Forward Primer (5’-3’) Reverse Primer (5’-3’)—product length

OT1—F-GTGCTGAAAAACCACTGGGG R-TCTGGGGGCAAAACATTGGT - 1207 bp. OT2 - F-TACTGGGCAACAGATGCTTGT R-AAAGTACCTGGAAGCCACCAA - 1496 bp. OT3—F-AGCAGAGGAGGGGAAGGTTT R-CAGATAGGTGCCTTCCCCAA - 1481 bp. OT4—F-CAGACATCCAAGGGACCACTG R-AACTGAAGCCAAGGGCACAG—1406 bp. OT5—F-TTGTCTTGGGGCCAGATCAC R-TCTGACCAGCCATGTTAGGC—1117 bp. OT6—F-TTGGAGCCAAATTATCTTGATTGCT R-TGTGTGGAATAGCCAGTCACC—1051 bp. OT7—F-AAAGCATCCCTCCCCAAACT R-ATCTCAGCTGACAAGGGCAAG—1362 bp. OT8—F-GTCAGTGGGGCTTAAGCTGT R-AGTACAGACTGGAGCCAGGA—1273 bp. OT9—F-TGCTGAAGGTCAAACGACCA R-AGCACAATCAGGTTGCCTCT—1460 bp. OT10—F-GGATCAGCTGTTGACCCCAA R-AGTAATGCTGAGACCGAGCC—1082 bp. The chosen clones were analyzed for karyotype using the NanoString nCounter Human Karyotype Panel according to the manufacturer´s protocol.

### Murine Lmna^H222P/H222P^ Model

Homozygous males *Lmna*^H222P/H222P^, previously characterized in,^16,21^ were used for this study. Mice were provided standard chow and water ad libitum and maintained in a disease-free barrier facility under controlled conditions: 12-hour light/dark cycle, a temperature of 22 °C, and 55% relative humidity. All animal experiments were approved by the French Ministry of Higher Education and Research and conducted at the Center for Research in Myology in compliance with the European Directive 2010/63/EU on the protection of animals used for scientific purposes (no. 47426-202402051349935). Study design, conduct, and reporting followed the ARRIVE 2.0 guidelines. Mice used in this study were 5 months of age, a stage at which the cardiac disease phenotype is already established as evaluated by echocardiography and histology. To evaluate the therapeutic efficacy of Simtuzumab, treatment was administered between 4 and 5 months of age, corresponding to a period when pathological features are present but not yet end stage. This timeframe was selected to allow assessment of the compound’s potential to modify disease progression and improve cardiac function under conditions that closely reflect the clinical scenario of therapeutic intervention rather than prevention.

### Cardiac Differentiation

Once hiPSC reached 90% confluency, 2-dimensional sandwich-based differentiation into ventricular-like hiPSC–cardiomyocytes (hiPSC-CMs) followed the Wnt pathway activation/inhibition protocol as described in.^22^ On day 9, the medium was changed to Roswell Park Memorial Institute 1640-B27. From day 9, hiPSC-CMs were further matured with 100 nmol/L 3,3’,5 triiodo-L-thyronine (T3; Sigma-Aldrich, catalog no. T2877) and 0.5 mmol/L N6,2′-O-Dibutyryladenosine 3′,5′-cyclic monophosphate sodium salt (N6,2′-O-dibutyryladenosine 3′,5′-cyclic monophosphate sodium salt; Sigma-Aldrich, catalog no. D0627).^23,24^ By day 20, hiPSC-CMs were purified using 4 mmol/L sodium L-lactate (Sigma-Aldrich, catalog no. L7022) in Silac medium (Gibco, catalog no. A2494201).^25^ All the experiments were performed between days 30 and 35 of cardiac differentiation. For the generation of engineered heart tissues (EHTs), hiPSCs were differentiated as described in Mosqueira et al^26^ or differentiated and generated as described in.^27^ hiPSC were differentiated into cardiomyocytes following a 3-step protocol with the generation of embryoid bodies in spinner flasks and cast into EHTs. In brief, EHTs were generated on silicone racks with hiPSC-CM suspensions in a 24-well format with 1×106 cells per EHT in a fibrin matrix consisting of 10% Matrigel (BD Biosciences, 256235), 5 mg/mL bovine fibrinogen (200 mg/mL; Sigma catalog no. F4753), aprotinin (Sigma, catalog no. A1153), 2× DMEM, 10 µmol/L Y-27632, and 3 U/mL thrombin (Biopur, catalog no. BP11101104). EHTs were kept at 37 °C at 7% CO_2_ and 40% O2, and medium (DMEM, 10% FCS, 0.1% insulin, 0.5% penicillin/streptomycin, 0.1% aprotinin) was changed on Mondays, Wednesdays, and Fridays. Contraction was monitored over time with a video-optical analysis system (EHT Technologies).

### Calcium Measurements

At day 23, contracting monolayer regions were enzymatically dissociated to isolate hiPSC-CMs. The cultures were rinsed twice with calcium- and magnesium-free PBS (Sigma, D8537), followed by incubation for 10 minutes at 37 °C in prewarmed TrypLE (Gibco, catalog no. 12604013) with gentle agitation. The reaction was quenched by adding Roswell Park Memorial Institute1640 supplemented with B27. Cell aggregates were centrifuged at 300 g for 10 minutes to obtain a pellet. The pellet was resuspended in Roswell Park Memorial Institute1640-B27 containing rho-associated coiled-coil-containing protein kinase inhibitor, and cells were seeded at a density of 10 000 cells/cm^2^ onto Matrigel hES-qualified coated dishes in Roswell Park Memorial Institute1640-B27 medium for 7 days before the experiments. Diastolic calcium levels were assessed using the ratiometric fluorescent Ca^2+^ sensor dye Indo-1-AM (Molecular Probes, catalog no. I1223). Enzymatically dissociated hiPSC-CMs were incubated for 25 min at 37 °C with 2 mmol/L Indo-1-AM in the Tyrode solution. The diastolic calcium level was measured using the IonOptix acquisition system FSI700 fluorescence with PMT400, under electrical pacing (1 Hz, 20 V, 5 ms duration). Diastolic time was defined as the interval between the time point of peak cytosolic Ca^2^^+^ (maximum Indo-1 am fluorescence ratio) and the point at which fluorescence returned to 90% of the resting (diastolic) baseline value, corresponding to the relaxation phase of the calcium transient. All ratios were measured using IonOptix Software—IonWizard 7.3. For measuring the sensitivity of EHT to external calcium concentrations, EHTs were transferred to DMEM with 1.8 mmol/L calcium for 30 minutes, washed twice with DMEM with 0.1 mmol/L calcium for an hour, and then measured under stepwise calcium concentration increase starting at 0.3 mmol/L until 3 mmol/L calcium and under electrical pacing (1.5 Hz, 2 V in biphasic pulses of 4 ms).

### Measurement of Contractile Properties by Video-Edge Capture

Enzymatically dissociated hiPSC-CMs were seeded in microscopy dishes (Ibidi, catalog no. 81156) containing 2 mL of 1.8 mmol/L to measure the contractile parameters under electrical pacing (1 Hz, 20 V, 5ms duration), using microscopy video-edge capture (63 frames/s, 25 s/positions, 15 ms/image), with 63× oil objective of Zeiss LSM900 and Zen Software at 37 °C. Acquisitions were analyzed using a MatLab homemade script as published before.^19,28,29^ For each video, an regions of interest along the cell border was tracked frame-by-frame to generate a displacement-versus-time trace reflecting auxotonic shortening and relaxation. Individual beats were automatically identified by peak detection. For each beat, the start of contraction was defined as the point at which displacement departed from baseline (threshold-based rise onset), the peak contraction corresponded to the maximal displacement, and the end of relaxation was defined as the subsequent displacement minimum. Contraction time was calculated as the interval from contraction onset to peak displacement, and relaxation time as the interval from peak displacement to the end of relaxation. Systolic and diastolic durations were derived accordingly, and values were averaged across all beats in each video. Heterogeneity values represented the beat-to-beat variability of each contractile parameter within a single video. For each video, all beats were analyzed individually, and the SD of the measured values was used as the heterogeneity index.

For EHT measurements, EHTs were kept in culture for up to 50 days, and measurement of contractile properties occurred as previously described via video optical recording and making use of CTMV software.

### Cellular Morphology Assessment

Enzymatically dissociated hiPSC-CMs were seeded in microscopy dishes (Ibidi, catalog no. 81156). Cells were fixed with 4% PFA solution for 15 minutes at room temperature (RT), permeabilized using 2 mL of permeabilization solution (1× PBS+0.01% Tritton 100×) for 10 minutes at RT, and incubated with 1 mL of blocking solution (1× PBS+1% BSA) for 30 minutes at RT. For immunostaining, cells were incubated 1 hour at RT or overnight at 4 °C with 500 mL of a solution of primary antibodies (1× PBS+0.1% BSA). Cells were incubated for 2 hours at RT protected from light, with secondary antibody solution (1× PBS+0.01% BSA). Image acquisition was performed using a 63× oil objective of the Zeiss LSM900 microscope and Zen Software. To measure the cell morphology and organizational parameters, images were analyzed using MorphoScript under MatLab as previously described.^30^

### Nuclear Morphology Assessment

Enzymatically dissociated hiPSC-CMs were fixed, permeabilized, and then marked with DAPI, following the same protocol as in the section Cellular Morphology Assessment. The images were acquired using a Nikon Ti2 microscope, equipped with a motorized plate and a 100× oil objective with a Prime 95B Scientific CMOS camera. The microscope was controlled by MetaMorph 7.10 (Molecular Devices) software with a Pixel resolution of 0.11 µm/px at 16 bits. Images were pretreated with FIJI/ImageJ^31^ software. The nucleus was segmented using CellPose^32^ v2.2.2 with a GPU NVIDIA GeForce RTX 3080 Ti, using the performed model cyto2 and a diameter fixed at 120 pixels. Segmentation masks were imported into QuPath^33^ v0.3.2, where nuclear parameters were measured. For nuclear analysis in EHT samples, EHTs were fixed in ROTIHistofix, permeabilized, and stained with Hoechst (Thermo Fisher Scientific, catalog no. 62249). Whole-mount EHTs were imaged in Z-stacks with a Zeiss LSM800 microscope and analyzed with Imaris (Bitplane) to measure nuclear length in 3D-reconstructed images.

### Functional and Morphological Heart Exploration

Mice were anesthetized with 0.75% isoflurane in O_2_ and placed on a heating pad (28 °C). Transthoracic echocardiography was performed using a Vivid 7 Dimension/Vivid7 PRO ultrasound (GE HealthCare) with an 11 MHz transducer applied to the chest wall. Cardiac ventricular dimensions and fractional shortening were measured in 2-dimensional mode and M-mode 3 times for the number of animals indicated. A blinded echocardiographer, unaware of the genotype and the treatment, performed the examinations. Electrocardiograms were recorded from mice using the noninvasive ecgTUNNEL (Emka Technologies) with minimal filtering. Waveforms were recorded using Iox2 Software (Emka Technologies), and intervals were measured manually with ecgAUTO (Emka Technologies) software. The electrocardiographer was blinded to the mouse genotype.

### Chromosome Painting Assay

Chromosome territories were visualized using the chromosome fluorescent in situ hybridization approach, known as chromosome painting. The probes used were whole-chromosome and coupled with green (505 nm) or orange (552 nm) fluorophores (MetaSystems Probes, catalog no. XCyting Chromosome Paint). The chromosome painting experiment was performed according to a combination of manufacturer’s and published protocols.^34^ Briefly, hiPSC-CMs were seeded in glass coverslips, and then fixed with 4% PFA for 20 minutes at RT, permeabilized with 0.05% Triton 100× for 20 minutes at RT, and depurinated with 0.1 mol/L HCl for 5 minutes at RT. Cells were washed twice with 2× SSC, 5 minutes at RT, and 50% formamide/4× SSC at 37 °C, 4 hours in a humidified chamber. Cells and probes were mixed on a slide, sealed with rubber cement (Talens, catalog no. 95306500), and denatured at 75 °C for 2 minutes. The hybridization step was performed at 37 °C, overnight in a humidified chamber. Cells were washed with 0.4× SSC at 75 °C, 2 minutes, 2× SSC+0.05% Tween-20, 30 s, and H_2_O to avoid crystallization. Cells were counterstained with DAPI for 10 minutes at RT, light-protected, and a coverslip was mounted in a glass slide. The chromosome organization was visualized using a Nikon Ti2 microscope coupled with a live super-resolution module (Live-SR 3D, Gataca Systems), equipped with a motorized plate, and coupled with a 100x oil objective with Prime 95B Scientific CMsOS camera. The acquisition was realized using MetaMorph 7.10 (Molecular Devices) software with a Pixel resolution of 0.0658 µm/px at 16 bits. Nuclei were manually cut out before acquisition to avoid bleaching of other nuclei. A pipeline was developed to measure the chromosome distribution in the nucleus. Nucleus masks were obtained using QuPath^33^ and binarized with ImageJ/Fiji.^31^ An algorithm was implemented in QuPath^33^ to divide each detected binarized nucleus mask into 10 bins. The bins from 1 to 5 correspond to the nuclear center, whereas bins from 6 to 10 correspond to the nuclear periphery. This approach allows for a detailed analysis of nuclear structure by measuring the average pixel fluorescence intensity within each bin in the fluorescein isothiocyanate and tetramethylrhodamine isothiocyanate channels. For each bin, the maximum pixel fluorescence intensity was calculated across all the acquired images for both mutant and control groups for a single probe. The normalized intensity per bin was calculated for each cell line, and each probe was determined using the minimum and maximum pixel fluorescence intensities. This data was then used to create heatmaps and graphs on Prism10.

### hiPSC-CMs RNA-Seq Analysis

Total RNAs were extracted from hiPSC-CMs and ventricular myocardial mouse tissues using the RNeasy Mini Kit Protocol (QiaGen, catalog no. 74104). Briefly, RNAs were extracted using Qiazol/chloroform solution and purified with columns. The integrity of the RNA extracts was analyzed on a Bioanalyzer (Agilent), with RNA integrity number readout of >8 being used. Strand-specific sequencing libraries were generated using the TruSeq stranded total RNA library preparation kit, including depletion of RNA (Illumina Inc), and sequenced on the NovaSeq6000 instrument (Illumina Inc) to give 40 million reads of 100 bp, paired-end, by sample. The library preparation and sequencing were performed by the iGenSeq platform at Institut du Cerveau (Paris). The RNA-seq results were obtained using the following pipeline: reads were preprocessed using the fastp^35^ module v0.20.0 and aligned on GRCh38 human or GRCMs39 mouse reference genome using Star^36^ v2.7.5a. Pairs of reads aligned were annotated using gtf annotation files provided by ENSEMBL. Genome-aligned reads were indexed with Samtools^37^ v1.13 and quantified with Qualimap^38^ v2.2.2b. The number of reads uniquely mapping coding RNA was compiled in a counting matrix using FeatureCounts^39^ v2.0.1. All steps were submitted to quality control using Mutliqc^40^ v1.13. Differential analysis was performed using the DESeq2^41^ package on R using a threshold of *P* adjusted value 0.05 and log2-fold change (log2FC) 0.5. Gene Ontology tables were obtained using Enrichr,^42–44^ STRING^45^ v12.0 (https://string-db.org), and the ggplot2^46^ package on RStudio. Transcriptomics data were available in the GEO database: GSE289418 (hiPSC-CMs) and GSE312730 (hiPSC-EHTs).

### Protein Extraction and Immunoblotting

Total proteins were isolated from cardiac sections using an extraction buffer (Cell Signaling) containing protease and phosphatase inhibitors (Thermo Scientific). The protein content of the samples was determined using the BCA Protein Assay Kit (Thermo Scientific, catalog no. 23227). Protein extracts (50 µg) were analyzed by NuPAGE 4% to 12% Bis-Tris gels and transferred to nitrocellulose membranes (Invitrogen). After washing with Tris-buffered saline containing 1% Tween 20 (TBS-T), the membranes were blocked with 5% BSA in TBS-T for 1 hour at RT and then incubated with the appropriate antibody at 4 °C, overnight. Membranes were incubated with fluorescent-conjugated anti-mouse or anti-rabbit secondary antibodies (Bio-Rad) for 1 hour at RT. Antibody detection was imaged using the ChemiDoc MP Imaging System (Bio-Rad) and ImageLab software (Bio-Rad). Quantification was performed using ImageLab software (Bio-Rad).

### Immunostaining of Cardiac Sections

Frozen heart tissue was cut into 10 µm-thick cryosections, fixed (10 minutes, 4% PFA in 1× PBS at RT), and blocked (1% BSA, 5% goat serum, 0.2% Triton X-100 in 1× PBS) for 1 hour at RT. Sections were incubated with antibodies (1% BSA, 5% goat serum, 0.2% Triton X-100 in 1× PBS), overnight at 4 °C, and then washed with 1× PBS. The sections were then incubated with the appropriate secondary antibodies for 1 hour before being washed with PBS. Nuclei were counterstained with 0.005% Hoechst in 1× PBS for 5 minutes at RT. The slides were mounted with Fluoromount-G (Invitrogen). Immunofluorescence microscopy was performed using an EVOS M5000 microscope (Invitrogen, catalog no. AMF5000), a fully inverted imaging system equipped with 4-color fluorescence, transmitted light, and imaging capabilities. Images were acquired at a 20× objective with a pixel resolution of 0.44 µm/px. Images were processed using FIJI/ImageJ^31^ software.

### Immunostaining of hiPSC-CMs Cultured in 2-Dimensional Cardiac Sheets

Immunocytochemistry was then conducted on intact cardiac sheets cultured for 30 days. Cells were fixed with 4% PFA solution for 20 minutes at RT, permeabilized and blocked using a solution (1× PBS+0.01% Triton 100×+1% BSA) for 30 minutes at RT. For immunostaining, cells were incubated 1h at RT or overnight at 4 °C with 500 mL of a solution of primary antibodies (1× PBS+0.1% BSA). Primary antibodies, including alpha-actinin (Sigma no. A7811) and collagen 1 type A1 (Cell Signaling no. 39952), were diluted 1:200 in 1% BSA. Cells were incubated for 2 hours at RT protected from light, with secondary antibody solution (1× PBS+0.01% BSA) and DAPI. Image acquisition was performed using a 63× oil objective of the Zeiss LSM900 microscope and Zen Software. To measure the COL1A1 (collagen 1A1) deposition, image analysis was performed using ImageJ/Fiji.^31^

### Electron Microscopy

The cardiac sections were fixed in 2.5% glutaraldehyde diluted in PBS for 1 hour at RT. After washing with PBS, samples were postfixed in 1% osmium tetroxide, dehydrated through a graded series of acetone, and embedded in epoxy resin. Ultrathin sections were cut to 90 nm, stained with uranyl acetate and lead citrate, examined with a JEM-1011 transmission electron microscope (JEOL Ltd, Tokyo, Japan), and photographed using an Erlangshen 1000 digital camera (GATAN) with Digital Micrograph software (GATAN). The isolated cardiomyocytes were fixed in 2.5% glutaraldehyde in PBS for 3 hours at room temperature. After PBS washes, 0.15 mol/L of cacodylate was introduced and incubated at 40 °C in a water bath. 3% of preheated agarose was added to the tube before solidification. The agarose-embedded cell pellet was cut into small pieces and incubated in a salt shaker with 0.15 mol/L cacodylate, followed by 2% osmium tetroxide for 60 minutes at room temperature, shielded from light. After washing with distilled water, samples were progressively dehydrated in ethanol baths, then incubated twice with propylene oxide for 10 minutes at room temperature, and finally incubated in various epon resin baths before flat mold polymerization for 48 hours at 50 °C. Ultra-thin sections (80 nm) were prepared using an EM UC7 ultramicrotome (Leica) and contrasted with 2% uranyl acetate and lead citrate. Primary observations were conducted with a transmission electron microscope operating at an acceleration voltage of 120 kV (JEM-1400Flash, JEOL) and a high-resolution digital camera (EM-SIS, Xarosa).

### Histological Analysis

Frozen hearts were cut into 8 µm thick sections and stained with Sirius red. Briefly, heart sections were fixed in 4% formaldehyde for 10 minutes, rinsed in EtOH 100%, dried for 20 minutes, and stained in 0.3% Sirius red solution for 1 hour. Then, sections were put in acetic acid 0.5% for 5 minutes twice, in EtOH 100% for 5 minutes 3×, and finally in xylene for 10 minutes twice. Collagen fibrils were detected in red, whereas cytoplasm remained yellow.

### RNA Isolation and Reverse-Transcription Quantitative Polymerase Chain Reaction

Total RNA was extracted from the mouse heart using the RNeasy isolation kit (Qiagen) according to the manufacturer’s instructions. The adequacy and integrity of the extracted RNA were determined with the 2100 Bioanalyzer system (Agilent) according to the manufacturer’s instructions. cDNA was synthesized using the SuperScript III First-Strand synthesis system according to the manufacturer’s instructions (Invitrogen, catalog no. 18080051). Real-time quantitative polymerase chain reaction was performed with SYBR Green I Master mix (Roche, catalog no. 04707516001) on the LightCycler 480 instrument (Roche). The primers used in this study are described in Table S1. The expression of each target gene in cardiac tissues from *Lmna*^*H222P/H222P*^ mice was compared with that of WT mice after being normalized to the housekeeping gene (RplpO). The ΔΔCT formula was used to compute the fold change relative to the control sample using the method prescribed.

### Anti-Loxl2 Treatment

Simtuzumab (anti-Loxl2; Selleckchem, catalog no. A2408) was administered by intraperitoneal injection to *Lmna*^*H222P/H222P*^ mice at a dose of 5 mg/kg, 3× a week for 1 month. *LMNA* H222P hiPSC-CMs cultivated in 2-dimensional cardiac sheets were treated at day 15 of differentiation with 1 µmol/L Simtuzumab (Selleckchem, catalog no. A2408) for 15 days, while control sheets received an equivalent volume of 1× PBS.

### Statistical Analysis

The statistical significance of functional experiments on hiPSC-CMs was analyzed using a nonparametric Mann-Whitney *U* test. Cardiac echocardiography experiments in mice were analyzed using 1-way ANOVA, with post hoc comparisons as appropriate. The statistical significance of the frequency, contraction time, and force in hiPSC-EHTs was analyzed using a Nested *t* test statistical test. The calcium EC50 experiment was analyzed using a nonparametric Mann-Whitney *U* test. Two-way ANOVA was used to analyze chromosome spatial organization, with post hoc tests as needed. mRNA fibrosis marker expression was analyzed using an unpaired Student *t* test. Loxl2 relative expression was quantified using an unpaired Student *t* test. The mean fluorescence intensity of LOXL2 immunostaining in hiPSC-CMs was quantified using an unpaired Student *t* test. The effect of Loxl2 treatment on cardiac function, as measured by echocardiography, was analyzed using 1-way ANOVA with Tukey multiple comparisons test. A significance level of *P*<0.05 was used for all statistical tests. Data are presented as the mean±SEM, and independent differentiation numbers are indicated in the figure legends. Statistical analyses were performed using Prism software (GraphPad v9).

## Results

### Modeling LMNA-Associated DCM In Vitro

**Figure 1.**
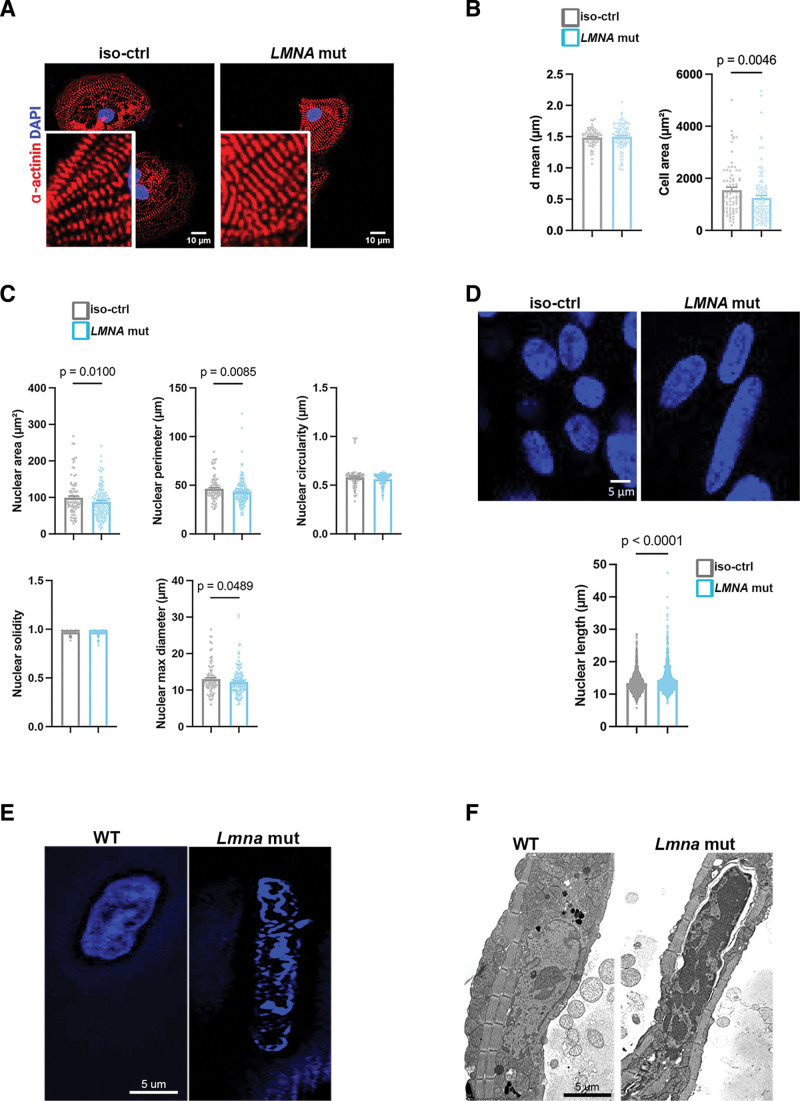
**Mutated A-type lamins caused cardiac structural impairments. A**, Representative immunostaining image of human induced pluripotent stem cells (hiPSC-CMs; *LMNA* p.H222P mutant hiPSC [*LMNA* mut] and of the corrected control isogenic [Ctrl-iso]) showing 𝛼-actinin labeling of sarcomeres. Nuclear counterstains are also shown. Scale bar 10 𝜇m. **B**, Mean distance between sarcomeres (dmean) and cell area in hiPSC-CMs (n_H222P_=3, n_corr.H222P_ =3). **C**, Nuclear structural parameters (nuclear area, nuclear perimeter, nuclear circularity, nuclear solidity, nuclear maximal diameter) of hiPSC-CMs (n_H222P_=2, n_corr.H222P_=2). **D**, **Top**: Representative nuclear-stained (Hoechst) images of hiPSC engineered heart tissues (EHTs). Scale bar 5 𝜇m. **Bottom**: Nuclear length of hiPSC-EHTs (n_H222P_=14, n_corr.H222P_=13). **E**, Fluorescence micrographs showing nuclei stained with DAPI of isolated cardiomyocytes from 5-month-old male wild-type (WT) and *Lmna*^H222P/H222P^ mice (*Lmna* mut). **F**, Electron microscopy image showing nuclei of isolated cardiomyocytes from 5-month-old male WT and *Lmna*^H222P/H222P^ mice. Statistics: (**B**, **C**, **E**) Mann-Whitney *U* test statistical test, mean±SEM.

We first employed the patient-specific *LMNA* p.H222P hiPSC line (*LMNA* H222P) and its isogenic control (*LMNA* corr.H222P) as previously published^18^ to explore the morphological consequences of the *LMNA* p.H222P mutation (Figure S1). The patient-specific hiPSCs were differentiated into ventricular-like hiPSC-CMs following a maturation protocol by small molecules as previously described.^23,24^ By day 30 of differentiation, we explored the cellular organization and contractile properties. Although *LMNA* H222P hiPSC-CMs exhibited normal sarcomere compaction compared with control cells (Figure 1A), their cell area was significantly reduced (Figure 1B). Additionally, mutated hiPSC-CMs displayed a decreased nuclear area and perimeter compared with controls (Figure 1C). No significant differences in nuclear circularity, solidity (indicative of invaginations), and nuclear maximal diameter were observed between *LMNA* H222P and *LMNA* corr.H222P hiPSC-CMs (Figure 1C). Furthermore, hiPSC-CMs were cast into EHTs and cultured for 30 to 50 days. The EHT format results in the maturation of cardiomyocytes and allows the measurement of tissue function.^47^ The *LMNA* H222P hiPSC-EHTs had more elongated nuclei than *LMNA* corr.H222P hiPSC-EHTs (Figure 1D). Consistent with the hallmark nuclear abnormalities associated with *LMNA*-associated DCM, we examined nuclear morphology in isolated cardiomyocytes from *Lmna*^*H222P/H222P*^ mice and observed elongated nuclei (Figure 1E and 1F).

**Figure 2.**
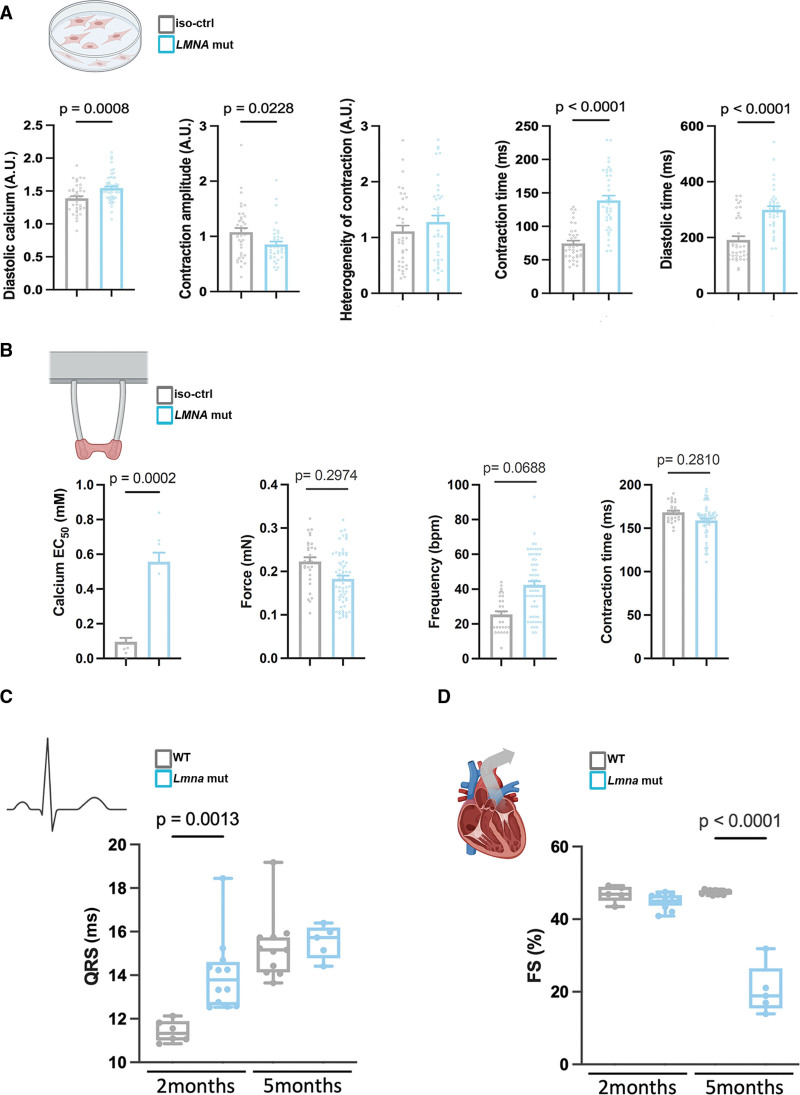
**Mutated A-type lamins caused cardiac functional defects. A**, Measurement of the ratio between the 2 emission wavelengths 405 nm and 480 nm under electrical pacing, reflecting the intracellular diastolic calcium concentration (n_H222P_=3, n_corr.H222P_=2), and the contractile parameters (n_H222P_=3, n_corr.H222P_=3) in *LMNA* H222P (*LMNA* mut) human induced pluripotent stem cells (hiPSC)-cardiomyocytes (CMs) compared with the corrected control isogenic (Ctrl-iso). The heterogeneity of contraction reflects the cell’s ability to contract homogeneously and is indicative of possible arrhythmias. The closer the value is to 1, the more cells contract simultaneously. The contraction and diastolic times correspond to the time for the cells to reach the peak and the bottom of the contraction, respectively. **B**, Alteration of sensitivity to external calcium concentration and contractile parameters in *LMNA* H222P hiPSC-EHTs (n_H222P_=69 engineered heart tissues [EHTs]/8 batches and n_corr.H222P_=30 EHTs/5 batches). **C**, QRS time duration measurement in *Lmna*^H222P/H222P^ mice. **D**, Fractional shortening measurement by echocardiography in *Lmna*^H222P/H222P^ mice. Statistics: (**A**) Mann-Whitney *U* test statistical test, mean±SEM, (**B**) nested *t* test, mean±SEM, (**C** and **D**) 1-way ANOVA statistical test, mean±SEM.

We next investigated excitation-contraction coupling in *LMNA* corr.H222P hiPSC-CMs. Intracellular calcium handling was monitored under electrical pacing. Notably, *LMNA* H222P hiPSC-CMs exhibited significantly elevated diastolic calcium levels compared with *LMNA* corr.H222P hiPSC-CMs (Figure 2A; Figure S2). Elevated diastolic calcium is commonly associated with cardiac arrhythmias and contractile dysfunction, both hallmarks of *LMNA*-associated DCM.^48^ This abnormal calcium handling may result from defects in calcium signaling pathways, which are crucial for proper cardiac muscle contraction and relaxation.^49,50^ To further assess contractile properties, we measured responses in hiPSC-CMs. *LMNA* H222P hiPSC-CMs consistently demonstrated disrupted 2D cardiac sheets, indicative of increased tissue fragility. These cells also exhibited reduced contraction amplitude (hypocontractility) compared with *LMNA* corr.H222P hiPSC-CMs (Figure 2A; Figure S3). The proportion of arrhythmic events was evaluated by assessing contraction heterogeneity; while no significant differences were observed, *LMNA* H222P hiPSC-CMs tended to display higher heterogeneity in contractions (Figure 2A). Both contraction and diastolic times were prolonged in *LMNA* H222P compared with *LMNA* corr.H222P hiPSC-CMs (Figure 2A).

*LMNA* H222P and *LMNA* corr.H222P hiPSC-EHTs were analyzed in a calcium concentration response curve under 1.5 Hz electrical pacing. *LMNA* H222P hiPSC-EHTs exhibited a higher mean effective calcium concentration (EC_50_) than *LMNA* corr.H222P hiPSC-EHTs (Figure 2B), indicating lower calcium sensitivity in *LMNA* mutants. Furthermore, *LMNA* H222P hiPSC-EHTs displayed a tendency towards lower force (hypocontractility) and higher frequency (Figure 2B) during their plateau phase in culture.

To evaluate our in vitro findings in an integrated model, we examined the *Lmna*^*H222P/H222P*^ mouse model at 2 and 5 months old, corresponding to the pre- and post-DCM stages.^21^ *Lmna*^*H222P/H222P*^ mice exhibited an abnormal ECG profile, which was characterized by a prolonged QRS interval without reaching significance (Figure 2C), which reflects subtle cardiac conduction defects. The contractile function was also compromised, as indicated by a decreased fractional shortening in *Lmna*^*H222P/H222P*^ mice compared with WT mice (Figure 2D).

### LMNA H222P hiPSC-CMs Altered Chromosome Positioning and ECM-Related Gene Expression

**Figure 3.**
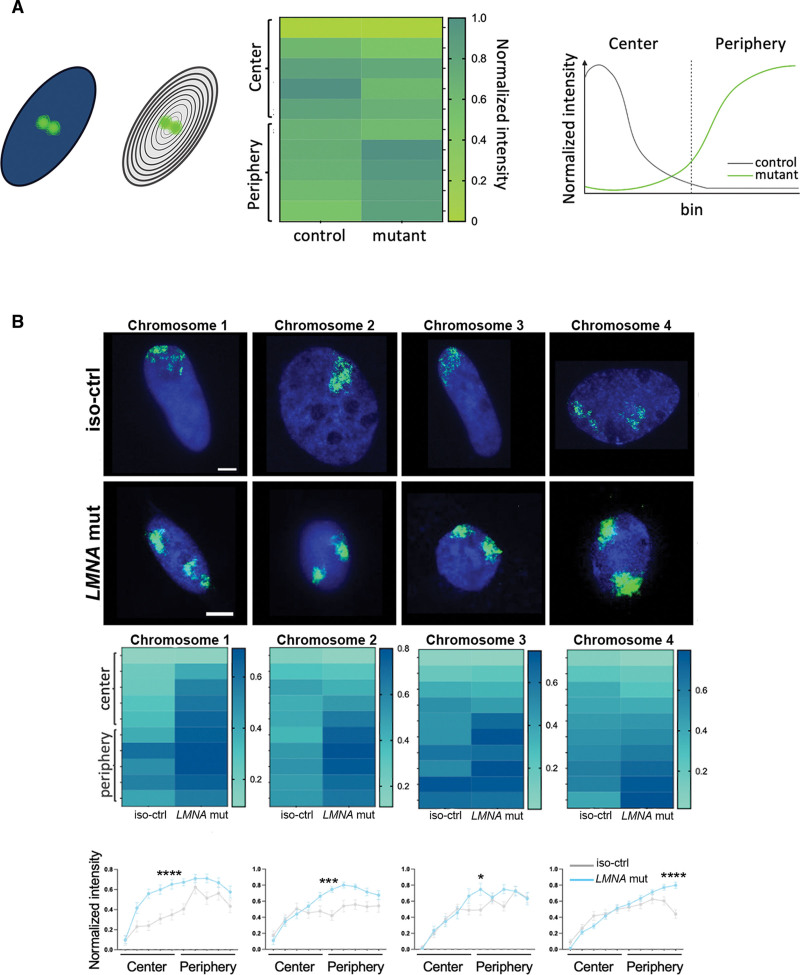
**Mutated A-type lamins altered the chromosome spatial organization. A**, Schematic pipeline of chromosome painting analysis. DAPI channel images were binarized to create nuclear masks, which were divided into 10 concentric rings (bins). Bins 1 to 5 correspond to the nuclear center, and bins 6 to 10 correspond to the nuclear periphery. The results are represented with heatmaps or graphs. **B**, **Top**: Representative images of chromosome painting experiments. Scale bar 5 𝜇m. **Bottom**: Heatmaps and graphs representing the results from the chromosome painting experiment (n_H222P_=2, n_corr.H222P_=2). Experiment (chr1, ^‡^ *P*=0.0001, ^‡^*P*=0.0006, ^‡^*P*=0.0003, ^†^*P*=0.0025; chr2, ^‡^*P*=0.0004, ^†^*P*=0.01, *P=0.0495; chr3, *P=0.0385; chr4, ‖*P*<0.0001). Statistics: 2-way ANOVA statistical test, mean±SEM.

**Figure 4.**
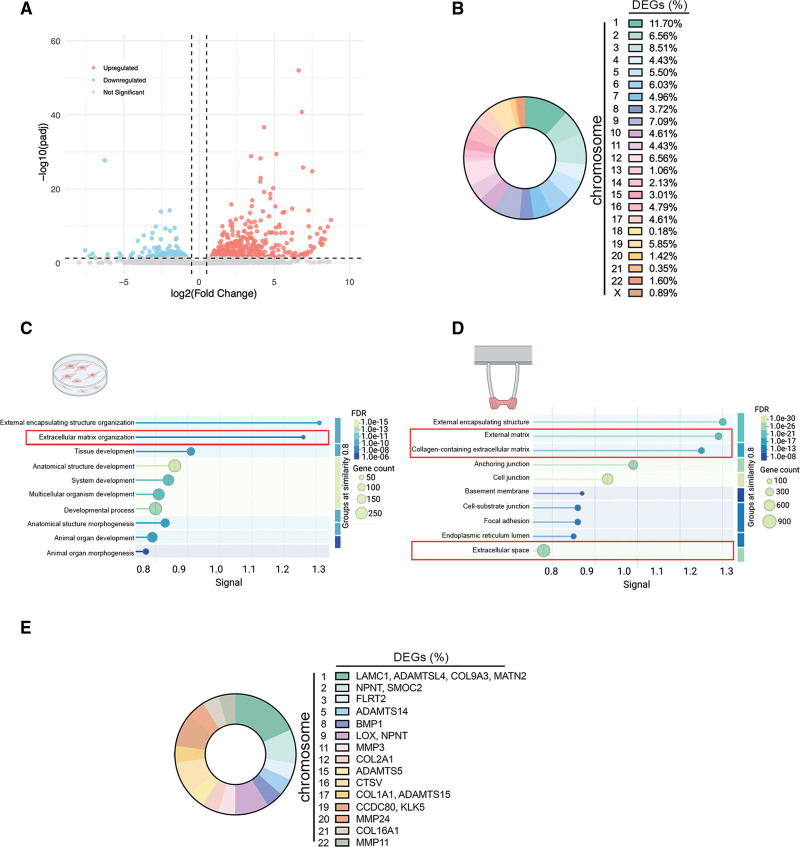
**Mutated A-type lamins lead to dysregulation of extracellular matrix gene expression. A**, Volcano plot representing DEGs between *LMNA* H222P (*LMNA* mut) and *LMNA* corr.H222P (Ctrl-iso) human induced pluripotent stem cells (hiPSC)–cardiomyocytes (CMs). The dotted lines correspond to the threshold used for the analysis. **B**, Graphical representation of differentially expressed genes (DEGs) percentage per chromosome in hiPSC-CMs. **C**, Graphical representation of the biological processes enrichment associated with DEGs in hiPSC-CMs. Generated with STRING v12.0. The size of each circle represents the number of genes associated with the GO term, and the color indicates the corrected *P* value (FDR). **D**, Graphical representation of the cellular component enrichment associated with DEGs in hiPSC–engineered heart tissues (EHTs). Generated with STRING v12.0. The size of each circle represents the number of genes associated with the gene ontology term, and the color indicates the corrected *P* value (false discovery rate). **E**, Graphical representation of extracellular matrix (ECM)-related DEGs per chromosome in hiPSC-CMs. RNA-seq analysis: n_H222P_=2, n_corr.H222P_=3; threshold, *P*adj 0.05, log2FC 0.5. Source data are accessible in GSE289418 for hiPSC-CMs RNA-seq and in GSE312730 for hiPSC-EHTs RNA-seq.

In eukaryotic cells, chromosomes occupy distinct territories within the nucleus, which is important for genome compaction and regulation.^51^ To assess the impact of the *LMNA* H222P mutation on chromosome organization, we performed chromosome painting and quantified their distribution in hiPSC-CMs (Figure 3A). We reported that the largest chromosomes (chr1, chr2, chr3, chr4) were significantly located towards the nuclear periphery in the *LMNA* H222P compared with *LMNA* corr.H222P hiPSC-CMs (Figure 3B; Figure S4). A-type lamins are essential for genome organization by regulating the distribution of LADs, which in turn influences gene expression.^51–54^ We next sought to identify abnormal expression of genes involved in the development of DCM caused by *LMNA* mutation using a bulk RNA-seq from hiPSC-CMs. This analysis yielded 607 differentially expressed genes (DEGs) between *LMNA* H222P and *LMNA* corr.H222P hiPSC-CMs (Figure 4A). We reported that the chromosomes with a spatial repositioning, as shown by chromosome painting (Figure 3A), exhibited a higher proportion of DEGs (Figure 4B). We next performed gene ontology analysis to identify the biological processes associated with the DEGs in *LMNA* H222P hiPSC-CMs. These DEGs were primarily involved in ECM organization (Figure 4C). We reported similar altered biological processes in *LMNA* H222P hiPSC-EHTs (GSE312730; Figure 4D). We observed that DEGs related to ECM organization were mostly present in chromosome 1 in *LMNA* H222P hiPSC-CMs (Figure 4E). To confirm the relevance of these findings in fully developed diseased hearts, we conducted a genome-wide RNA expression analysis on heart tissues from *Lmna*^*H222P/H222P*^ mice. This analysis revealed 3487 DEGs (Figure S5A and S5B) primarily associated with mitochondrial metabolism and ECM organization (Figure S5C). The dysregulation of these genes could contribute to pathological processes such as fibrotic remodeling and electrical conduction defects, both of which are hallmarks of DCM caused by *LMNA* mutations.^55^ A comparison of biological processes linked to the DEGs in both in vitro and in vivo models revealed a common focus on ECM structure, organization, and remodeling (Figure S5D).

### Loxl2 Mediates DCM Caused by LMNA Mutation

**Figure 5.**
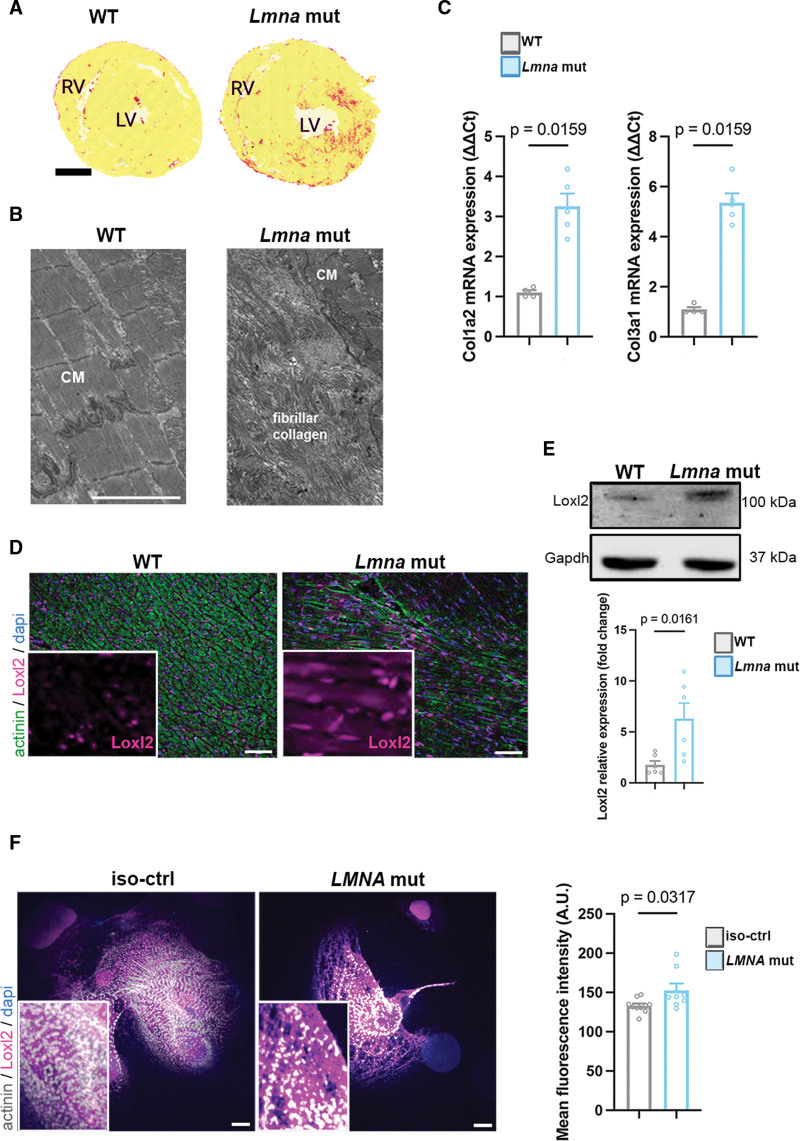
**Mutated A-type lamins lead to an increase in Loxl2 protein expression. A**, Transverse cardiac section from *Lmna*^H222P/H222P^ (*Lmna* mut) and wild type (WT) mice stained with Sirius red. Scale bar 1 mm. **B**, Representative electron microscopy images of fibrous collagen cardiac section from WT and *Lmna*^H222P/H222P^ mice. Scale bar 5 𝜇m. **C**, Gene expression of *Col1a2* and *Col3a1* in cardiac tissue from WT and *Lmna*^H222P/H222P^ mice. **D**, Representative immunostaining images of Loxl2 in cardiac sections from WT and *Lmna*^H222P/H222P^ mice. Scale bar 100𝜇m. **E**, Immunoblot showing Loxl2 and Gapdh expression in hearts of WT and *Lmna*^H222P/H222P^ mice. Bar graph showing the quantification of Loxl2 (n_Lmna_=2, n_WT_=2). **F**, Representative immunostaining of Loxl2 protein expression in hiPSC-CMs. Scale bar 10 𝜇m. Bar graph representing the quantification of the mean fluorescence of Loxl2 protein expression. Statistics: (**C**) Mann-Whitney *U* test statistic, mean±SEM; (**E** and **F**) unpaired *t* test, mean±SEM.

The role of ECM remodeling in DCM caused by *LMNA* mutations remains underexplored. However, we observed significant fibrosis development in hearts from *Lmna*^*H222P/H222P*^ mice compared with WT mice, as demonstrated by histology (Figure 5A) and electron microscopy (Figure 5B). These findings were further supported by elevated *Col1a2* and *Col3a1* mRNA expression in *Lmna*^*H222P/H222P*^ mice compared with WT mice (Figure 5C), both of which are established biomarkers of cardiac diseases, including heart failure.^56^

Among the DEGs related to ECM remodeling in hearts from *Lmna*^*H222P/H222P*^ compared with WT mice (Figure S6A and S6B), we focused on *Loxl2*, as it has been previously reported to be upregulated in failing hearts from mice and linked to increased interstitial fibrosis and cardiac dysfunction.^57^ Bulk RNA-seq data confirmed the upregulation of *Loxl2* in the hearts from *Lmna*^*H222P/H222P*^ mice compared with WT mice, with a similar trend observed in *LMNA* H222P hiPSC-CMs (GSE289418). We next sought to correlate changes in *Loxl2* gene expression with Loxl2 protein overexpression in vivo. We performed immunostaining on cardiac sections from *Lmna*^*H222P/H222P*^ mice. Loxl2 was upregulated in the mutated mice compared with WT mice (Figure 5D). Immunoblot experiments further validated these findings (Figure 5E; Figure S6). We observed a similar increased LOXL2 protein by immunostaining in *LMNA* H222P compared with *LMNA* corr.H222P hiPSC-CMs (Figure 5F). Our results are aligned with previous Omics data generated by us and others, showing that Loxl2 expression was upregulated in hiPSC-CMs, mouse, and patients, all carrying A-type lamins mutants (Figure S5).

**Figure 6.**
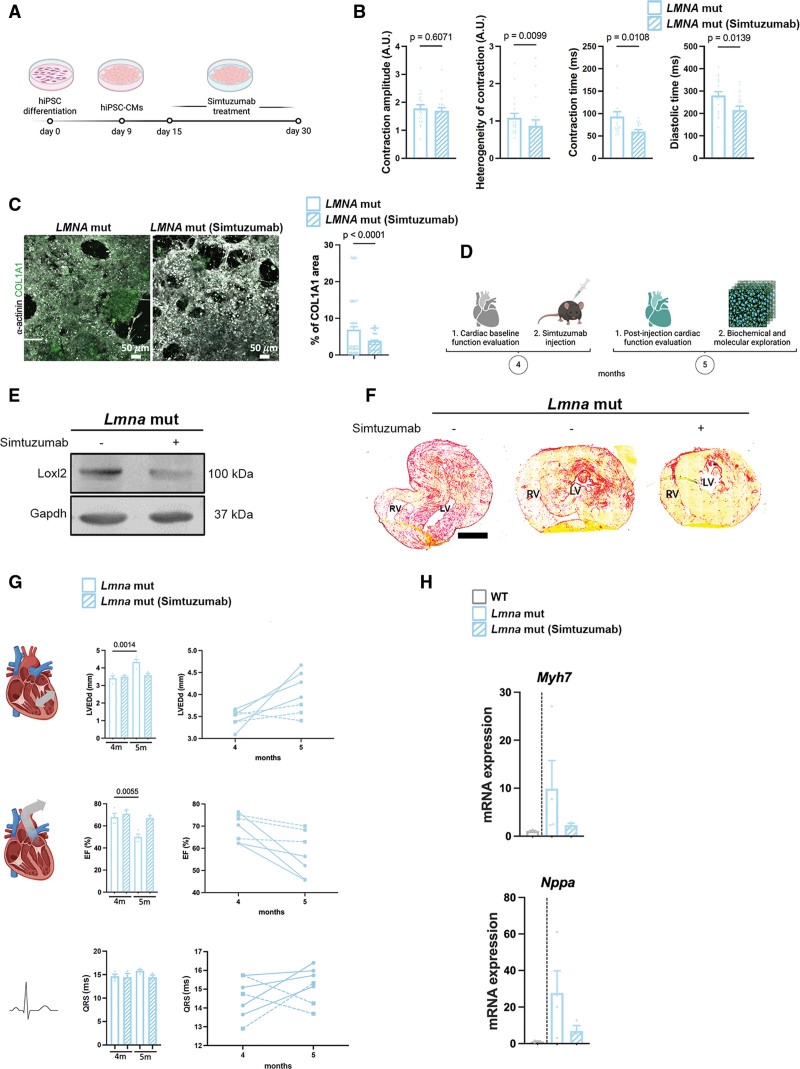
**Simtuzumab preserved cardiac function and prevented fibrosis both in vivo and in vitro. A**, Schematic representation of Simtuzumab human induced pluripotent stem cells (hiPSC)–cardiomyocytes (CMs) treatment. Generated with BioRender. **B**, Effect of Simtuzumab treatment on contractile parameters (contraction amplitude, heterogeneity of contraction, contraction time, and diastolic time) in *LMNA* H222P hiPSC-CMs. **C**, Effect of Simtuzumab treatment on COL1A1 deposition area in *LMNA* H222P hiPSC-CMs. Scale bar 50 𝜇m. **D**, Schematic representation of Simtuzumab mice treatment. Generated with BioRender. **E**, Immunoblot showing Loxl2 and Gapdh expression in hearts of *Lmna*^H222P/H222P^ mice (*Lmna* mut) treated or not with Simtuzumab. **F**, Transverse cardiac sections of *Lmna*^H222P/H222P^ mice treated or not with Simtuzumab, stained with Sirius red. **G**, Echocardiography (left ventricular end diastolic [LVEDd] and ejection fraction [EF]; mean±SEM) of *Lmna*^H222P/H222P^ mice with or without treatment, along the duration of the Simtuzumab treatment (n_Lmna+Simtuzumab_=3, n_Lmna no treat._=4). Illustrations generated with BioRender. **H**, Bar graph representing the mRNA relative expression of *Myh7* and *Nppa* (mean±SEM). Statistics: (**B**) Mann-Whitney *U* test statistic test, mean±SEM; (**C**) unpaired *t* test, mean±SEM; (**E**) Mann-Whitney *U* test statistic test, mean±SEM; (**G**) 1-way ANOVA statistic test, mean±SEM. **H**, Mann-Whitney *U* test statistic, mean±SEM.

To further decipher the role of Loxl2 in the development of DCM caused by *LMNA* mutations, we performed a pilot study of antibody-mediated Loxl2 inhibition in *LMNA* H222P hiPSC-CMs and *Lmna*^*H222P/H222P*^ mice using Simtuzumab^58^ (Figure 6A). In *LMNA* H222P hiPSC-CMs, Simtuzumab treatment decreased the heterogeneity of contraction and the contraction and diastolic times compared with the untreated control, whereas contraction amplitude remains unchanged (Figure 6B). We observed a reduction in the area of COL1A1 deposition in *LMNA* H222P hiPSC-CMs treated with Simtuzumab compared with the untreated control (Figure 6C). To evaluate these in vitro findings, we treated *Lmna*^H222P/H222P^ mice with Simtuzumab. Cardiac function and morphology were evaluated by echocardiography during the 1-month treatment duration (Figure 6D). Simtuzumab treatment decreased Loxl2 protein expression in *Lmna*^H222P/H222P^ mice compared with untreated mice (Figure 6E). Simtuzumab has been reported to reduce fibrosis in other disease models.^58^ We observed a reduction in fibrosis following treatment compared with untreated *Lmna*^H222P/H222P^ mice (Figure 6F). Furthermore, Simtuzumab treatment reduced left-ventricular dilatation in hearts from *Lmna*^H222P/H222P^ mice and improved left ventricular function (Figure 6G; Table). Although untreated *Lmna*^*H222P/H222P*^ mice exhibited progressive cardiac dysfunction and left ventricular dilatation between 4 and 5 months, these parameters remained stable in treated *Lmna*^*H222P/H222P*^ mice during the same period (Figure 6G). Notably, each treated *Lmna*^*H222P/H222P*^ mouse showed a stabilization of these parameters over time between 4 and 5 months, in contrast to their deterioration observed during the same period in untreated *Lmna*^*H222P/H222P*^ mice (Figure 6G). However, Simtuzumab treatment did not result in a statistically significant change in cardiac conduction defects, as measured by the QRS interval, in *Lmna*^*H222P/H222P*^ mice, although a trend toward improvement was observed (Figure 6G). Further, we showed that Simtuzumab lowered *Myh7* and *Nppa* gene expression in *Lmna*^H222P/H222P^ mice compared with untreated mice (Figure 6H). Together, these results suggest that inhibition of Loxl2 with Simtuzumab functionally preserves cardiac function in a mouse model of *LMNA*-associated DCM.

**Table.**
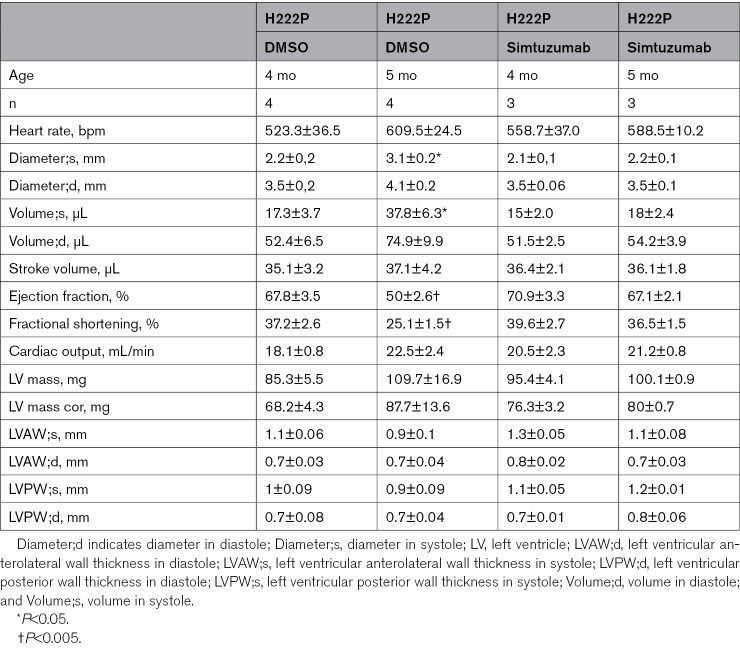
Echocardiography Data of Hearts From *Lmna*^H222P/H222P^ Mice at 4 and 5 Months, Treated or Untreated With Simtuzumab

## Discussion

In this study, we successfully modeled *LMNA*-associated DCM in vitro using a patient-specific *LMNA* H222P hiPSC line and its isogenic control, complemented by an in vivo *Lmna*^H222P/H222P^ mouse model. Our findings provide critical insights into the cellular, molecular, and functional alterations associated with DCM caused by *LMNA* mutations, highlighting the role of ECM remodeling in disease pathogenesis.

Our results provide the first-ever evidence that the *LMNA* mutation has a significant impact on chromosome positioning within the nucleus. This alteration in chromosome positioning suggests a disruption in the organization of the chromatin, which is normally tightly regulated by the nuclear lamina. The spatial remodeling observed could reflect impaired interactions between the A-type lamins and the LADs, regions of the genome that are anchored to the nuclear periphery and are critical for maintaining both the structural integrity of the nucleus and the proper organization of the genome. LADs play a pivotal role in gene regulation, as they influence the expression of genes by positioning them close to repressive or activating regions of the nuclear landscape. Disruptions in lamin A/C-LADs interactions could therefore lead to significant alterations in gene expression patterns, with potentially detrimental consequences for cellular function. Investigating Loxl2’s positioning within the nuclear landscape could also provide a deeper understanding of how alterations in the nuclear envelope and chromatin organization in diseases might influence gene expression and cellular function. This exploration could lead to the identification of novel therapeutic targets or biomarkers for diseases associated with LADs disruption and nuclear architecture defects. Ultimately, studying Loxl2 in the context of LADs could offer new perspectives on the molecular mechanisms linking chromatin organization, gene expression, and cellular pathology in disease conditions.

We further established that *LMNA*-associated DCM is associated with impaired ECM gene regulation, confirming previous work.^59–62^ In the context of heart failure, excessive ECM deposition leads to increased myocardial stiffness and aberrant signal transmission to cardiomyocytes. These changes ultimately result in impaired contractility.^63^ These findings highlight the critical role of ECM regulation in the pathogenesis of *LMNA*-associated DCM. A key finding of this study is the identification of *Loxl2* as significantly upregulated in *LMNA*-associated DCM. Loxl2 has been identified as a biomarker of heart failure.^57^ In mouse models of heart failure^57^ or diabetic cardiomyopathy,^64^ upregulation of Loxl2 promotes interstitial fibrosis, leading to cardiac dysfunction. We used a translational pharmacological approach by treating the mouse model with Simtuzumab to inhibit LOXL2 activity, thereby adding clinical relevance to the study. However, to strengthen these findings, they should be supported by an orthogonal genetic strategy, such as Loxl2 knockdown using shRNA, to rule out potential off-target or antibody-specific effects. Combining pharmacological inhibition with genetic silencing would increase confidence in the reported effects on cardiac structure and function. We further showed that inhibiting Loxl2 expression using Simtuzumab preserved cardiac function and morphology. These findings showed that Loxl2 contributes to the fibrotic remodeling and contractile dysfunction characteristic of *LMNA*-associated DCM and may represent a potential therapeutic target. Our results are consistent with the work of others. Notably, inhibition of Loxl2, either through antibody-mediated blockade, genetic deletion, or siRNA, significantly mitigates interstitial fibrosis and improves cardiac function.^57,64^ Loxl2 is notably upregulated by TGF-β, inflammation, mechanical stimuli, and hypoxia in endothelial and vascular smooth muscle cells.^57,65–67^ These factors are critical to cardiac homeostasis and pathogenesis, suggesting they could similarly contribute to Loxl2 upregulation in the heart. Understanding these mechanisms provides insight into Loxl2’s role in cardiac remodeling and its potential as a therapeutic target.

Given Loxl2’s involvement in matrix biology and its role in cellular processes, such as fibrosis, wound healing, and cancer progression, understanding whether it resides within an LAD in disease states could reveal new insights into its regulation and potential misregulation in pathological conditions. If Loxl2 is indeed found within an LAD in *LMNA*-associated DCM, it could suggest that its expression is controlled by the spatial organization of the genome, potentially highlighting a link between nuclear architecture and the regulation of genes involved in ECM remodeling.

Although Loxl2 has been involved in other conditions, including heart failure^57^ and diabetic cardiomyopathy,^64^ this study marks the first time Loxl2 has been associated with *LMNA*-associated DCM. Consequently, Loxl2 and its gene regulatory network emerge as promising therapeutic targets for enhancing cardiac function in DCM caused by *LMNA* mutations. Our comprehensive characterization has revealed crucial insights, highlighting the essential role of Loxl2 as a promising therapeutic target for *LMNA*-associated DCM. These findings pave the way for the development of effective treatments for this devastating disease.

## Limitations of the Study

Although our study provides valuable insights, certain limitations should be acknowledged. The patient-derived cardiomyocyte model, while powerful, may not fully recapitulate the complexity of adult cardiomyocytes or the chronic disease state observed in patients. We used a corrected isogenic control hiPSC line to evaluate the consequences of the *LMNA* H222P mutation. This strategy is currently the best for assessing the genotype/phenotype relationship with minimal polymorphism. However, isogenic control hiPSC lines may not always behave like unaffected healthy control hiPSC lines. Additional healthy control lines would strengthen our results.

Only male mice were used in this study due to the greater severity and earlier onset of the disease phenotype observed in males. Although female mice develop a comparable cardiac phenotype over time, their slower disease progression can complicate the assessment of early-stage mechanisms and therapeutic efficacy within the experimental timeframe. Nevertheless, it would be highly informative to extend these investigations to female mice in future studies. Evaluating the effects of Simtuzumab treatment in both sexes would provide a more comprehensive understanding of its therapeutic potential and ensure that the observed benefits are representative of the broader population, accounting for possible sex-specific differences in disease manifestation and drug response.

hiPSC-CMs offer a valuable model to study cardiac biology, but their use for investigating chromatin organization presents significant limitations. One of the primary challenges is that hiPSC-CMs are relatively immature compared with adult cardiomyocytes, both structurally and functionally. This immaturity extends to their chromatin architecture, which may not fully recapitulate the higher-order organization and gene regulatory landscape characteristic of adult heart cells. Consequently, findings derived from hiPSC-CMs may not accurately reflect chromatin dynamics in mature cardiomyocytes. Furthermore, our attempts to perform chromosome painting experiments in hiPSC-CMs were unsuccessful, preventing a direct comparison of chromosomal organization between these cells and their adult counterparts. This technical limitation further constrains our ability to draw definitive conclusions about the relevance of hiPSC-CMs for modeling chromatin architecture in the adult heart.

Additionally, the in vivo effects of Loxl2 inhibition were evaluated in a pilot study for a short period, and long-term studies are needed to assess sustained therapeutic benefits. This brief period may account for the modest Simtuzumab-mediated reduction of Loxl2 protein expression. Nevertheless, we observed preservation of cardiac function along with a reduction of cardiac fibrosis and left-ventricular dilatation, even with limited Loxl2 inhibition over a short treatment duration. These promising findings highlight the potential of Loxl2 as a therapeutic target and pave the way for the development of more potent Loxl2 inhibitors.

## ARTICLE INFORMATION

### Acknowledgments

The authors thank the genomic facility iGenSeq at the Brain Institute, Paris, for the RNA-seq libraries sequencing. We thank Dr Ben Yaou (Institute of Myology, Paris) for providing the fibroblasts from a biopsy of a patient carrying the *LMNA* p.H222P mutation. The authors thank Z. Guesmia from MyoImage, Myology Institute, Paris, for his help with image analysis and pipeline development. The authors thank Dr Monika Stoll from the University of Münster for the RNA sequencing of human induced pluripotent stem cells-engineering heart tissues.

### Author Contributions

Drs Muchir and Meli contributed to the conceptualization of the study. Drs Kervella, C. Peccate, Dr Grandi, and Dr Behrens contributed to the investigation. Cardiomyocyte isolation was performed by Drs Forand and Kervella. Cardiac function assessment was performed by Drs Mougenot and Muchir. Human induced pluripotent stem cells related experiments were performed by Drs Kervella, Behrens, Charrabi, and Meli. Image analysis and pipeline development were performed by Drs Guesmia, Kervella, and Behrens. Electron microscopy was performed by G. Brochier. Data analysis was performed by Drs Kervella, Singh, and Behrens. Writing—original draft was prepared by Drs Muchir, Meli, and Kervella. Writing—review and editing was prepared by Drs Kervella, Muchir, Meli, Andriantsitohaina, Behrens, Singh, and Eschenhagen. Funding acquisition was secured by Drs Muchir, Meli, and Eschenhagen. Supervision was provided by Drs Muchir and Meli.

### Sources of Funding

### Disclosures

None.

### Supplemental Material

Supplemental Methods

Tables S1

Figures S1–S6

Uncropped gels

## Supplementary Material

**Figure:**