# Supplementary material for: Simtuzumab Attenuates Loxl2-Mediated Extracellular Matrix Remodeling and Preserves Cardiac Function in LMNA Mutation-Induced Dilated Cardiomyopathy
Source: Circ Heart Fail. 2026 Mar 17;19(4):e013806. doi: 10.1161/CIRCHEARTFAILURE.125.013806 (PMC13095067; doi:10.1161/CIRCHEARTFAILURE.125.013806)
Supplement: Supplementary file 1 [file hhf-19-e013806-s001.pdf]

ARRIVE study plan

Please fill in all sections of the ARRIVE study plan.

Study details

Study title:	Echocardiography measurement to evaluate therapetutic effect of tretaments on Lmna p.H222P (C57Bl/6J) mice	Grant code:	n/a
Start date:	02/09/2024	End date:	01/10/2026
Project licence or permit number:	#47426-202401051349935	Project lead:	MUCHIR Antoine (antoine.muchir@inserm.fr)
Protocol numbers:	Echocardiography	Expected severity:	Low
Primary responsible:	MUCHIR Antoine	Contact details:	antoine.muchir@inserm.fr
Secondary contact:	n/a	Contact details:	n/a

Experimental animals

Species	Strain/Genotype	Sex	Age	Weight	Source	Number
Mouse	Lmna p.H222P (C57Bl/6J)	Male	4 to 5 weeks	22g	PMID: 31341969	7
					Total number	7

Experimental procedures

What is done and how is it done, when and how often.

Procedures:	Echocardiography
Surgical procedures:	n/a
Anaesthesia:	0.75% isoflurane in O2 and placed on a heating pad (28 °C)
Analgesia:	n/a

Locations:	Animal facility UMS28
Acclimatisation period:	n/a

Animal care and monitoring

Adverse events:	Anesthesia may induce thermal stress and, in rare cases, complications such as cardiorespiratory arrest.		
Humane endpoints:	The duration of an echocardiography examination is 5 to 5 minutes per mouse. Euthanasia will be performed at the end of the protocol using a regulatory-approved method for the purpose of tissue collection for analysis.		
Welfare monitoring:	Cardiac muscle impairment evidenced by a fractional shortening below 15% (monthly assessment by echocardiography). Respiratory distress related to anesthesia. In the event of respiratory distress, the oxygen percentage will be increased. If the condition persists, the animal will be euthanized.	Attached clinical assessment form:	No
Changes in housing and husbandry:	n/a		
Restrictions in veterinary care:	n/a		

Risks

Emergency procedures:	n/a
Potential risk to personnel:	n/a

Personnel involved in the experiment

MUCHIR Antoine	Animal handling	Trained and competent☒
MOUGENOT Nathalie	Echocardiography	Trained and competent☒

Study design and sample size

Experimental groups:	Lmna p.H222P (C57Bl/6J) versus treated Lmna p.H222P (C57Bl/6J)		
Experimental unit:	7	Sample size per group:	4
Justification for sample size:	The experimental procedures was designed to use a limited number of mice while allowing for robust statistical analysis. The experimental design for this project was determined using the IPSUR module of the R software, a program dedicated to statistical analysis. The significance threshold (alpha) is set at $\alpha = 0.05$, and the statistical power (beta) is set at $\beta = 0.8$. These criteria allow calculation of the minimum number of animals per group required to obtain quantitative data suitable for statistical analyses.		
EDA read only diagram:	n/a	Access code:	n/a

Inclusion and exclusion criteria

Inclusion criteria:	Genotype, sex
Exclusion criteria:	Animals or data points were removed from the study or analysis if predefined humane endpoints are reached, if animals die from causes unrelated to the experimental intervention, or if severe technical issues occur (e.g. failed imaging, poor-quality echocardiographic recordings). Data were be excluded if protocol deviations or technical artifacts prevent reliable measurement of the primary outcome. All exclusions were documented and reported transparently.
Expected attrition:	n/a

Randomisation and blinding/masking

Method of allocation to group:	Mice were randomly assigned to groups using a computer-generated randomisation sequence (R software). Randomisation was performed after genotyping and baseline assessment to ensure balanced groups. Investigators involved in data collection and analysis were blinded to group allocation whenever feasible.
Strategy to minimise confounders:	Systematic differences between groups were minimised by randomising cage placement within the animal facility and regularly rotating cages between rack positions. The order of treatments, procedures, and outcome assessments were randomised across groups. All animals were housed under identical environmental conditions and handled using standardized protocols. Whenever feasible, investigators performing treatments and analyses were blinded to group allocation.

Blinding strategy:	Group identity was concealed during allocation by using unique animal identification codes independent of treatment assignment. During study conduct, treatments were prepared and administered by personnel not involved in outcome assessment. Investigators performing functional assessments were blinded to group allocation. Data analysis was conducted using coded datasets, with group identities revealed only after analyses are completed.

Outcome measures and statistical methods

Outcome measures:	Echocardiography parameters
Primary outcome measure:	The experimental procedures will be designed to use a limited number of mice while allowing for robust statistical analysis. The experimental design for this project was determined using the IPSUR module of the R software, a program dedicated to statistical analysis. The significance threshold (alpha) is set at $\alpha = 0.05$, and the statistical power (beta) is set at $\beta = 0.8$. These criteria allow calculation of the minimum number of animals per group required to obtain quantitative data suitable for statistical analyses.
Analysis plans:	Cardiac echocardiography experiments in mice were analyzed using one-way ANOVA, with post-hoc comparisons as appropriate. Statistical analyses were performed using Prism software (GraphPad v9).

Sign off

Primary responsible:	MUCHIR Antoine	06/01/2026	☒ I confirm I am aware of my responsibilities as the primary responsible (e.g. conditions of the personal licence) and my training and competency record is up to date.
Project lead:	MUCHIR Antoine	06/01/2026	☒ I confirm I am aware of my responsibilities as a project lead/licence holder and this work is in line with the project.
Internal sign off:	MOUGENOT Nathalie	07/01/2026	
