# Supplementary material for: Simtuzumab Attenuates Loxl2-Mediated Extracellular Matrix Remodeling and Preserves Cardiac Function in LMNA Mutation-Induced Dilated Cardiomyopathy
Source: Circ Heart Fail. 2026 Mar 17;19(4):e013806. doi: 10.1161/CIRCHEARTFAILURE.125.013806 (PMC13095067; doi:10.1161/CIRCHEARTFAILURE.125.013806)

SUPPLEMENTAL MATERIALS

SUPPLEMENTAL METHODS

REAGENT or RESOURCE	SOURCE	IDENTIFIER
Antibodies		
Mouse monoclonal IgG1 anti-α-actinin	Sigma-Aldrich	Cat#A7732
Mouse recombinant IgG2b anti-cardiac Troponin I	Hytest	Cat#4T21
Rabbit polyclonal IgG anti-Loxl2	Abcam	Cat#ab96300
Mouse monoclonal IgM anti-sarcomeric α-actin	Sigma-Aldrich	Cat#A2172
Mouse monoclonal IgG1 anti-GAPDH (6C5)	Santa Cruz	Cat#sc322331
Goat anti-mouse IgG1 AlexaFluor 647	Invitrogen	Cat#A21240
Goat anti-mouse IgG2b AlexaFluor 555	Invitrogen	Cat#A21247
Goat anti-rabbit IgG AlexaFluor647	Thermo Fisher Scientific	Cat#A21244
Goat anti-mouse IgM cross-absorbed AlexaFluor546	Thermo Fisher Scientific	Cat#A21045
Goat anti-rabbit IgG StarBright Blue 520	Bio-Rad	Cat#12005870
Goat anti-mouse IgG StarBright Blue 700	Bio-Rad	Cat#12004162
Chemicals, peptides, and recombinant proteins		
DAPI (4',6-diamidino-2-phenylindol)	Thermo Fisher Scientific	Cat#10374168
Hoechst	Invitrogen	Cat#H3569
Fluoromount-G®	Invitrogen	Cat#00-4958-02
Anti-Loxl2 antibody (Simtuzumab)	Selleckchem	Cat#A2408
StemFlex Basal Medium	Gibco	Cat#A3349401
RPMI Medium 1640 (1X)	Gibco	Cat#21875091
SILAC RPMI 1640 Flex Media	Gibco	Cat#A2494201
Knockout™ Serum Replacement	Fisher Scientific	Cat#10828010
3,3',5 triodo-L-thyronine (T3)	Sigma-Aldrich	Cat#T2877
N6,2′-O-Dibutyryladenosine 3′,5′-cyclic monophosphate sodium salt (dAMPc)	Sigma-Aldrich	Cat#D0627
Sodium L-lactate	Sigma-Aldrich	Cat#L7022
Matrigel® hESC-Qualified Matrix, LDEV-free	Corning	Cat#354277
Matrigel® Growth Factor Reduced (GFR) Basement Membrane Matrix, *LDEV-free	Corning	Cat#354230
C59 Wnt antagonist	Abcam	Cat# ab142216
Chir99021	Sigma-Aldrich	Cat# SML1046
Y27632 Rock inhibitor	TargetMol	T1870
Indo-1-AM	Molecular Probes	Cat#I1223
Chromosome painting probes	MetaSystems Probes	Cat#XCyting Chromosome Paint
Rubber cement	Talens	Cat#95306500
Phosphate-buffered saline (DPBS, 1X), Dulbecco's formula, without calcium, without magnesium	Thermo Scientific	Cat#15434695
TrypLE™ Express Enzyme (1X), 500 mL, no phenol red	Fisher Scientific	Cat# 10718463
UltraPure™ DNase/RNase-Free Distilled Water	Invitrogen	Cat#10977015
D(+)-glucose anhydre	Fisher Scientific	Cat#10141520
Bovine Serum Albumin	Sigma-Aldrich	Cat#A9418
Pluronic™ F-68 Non-ionic Surfactant (100X)	Fisher Scientific	Cat#24040032
Gentamycin solution	Sigma-Aldrich	Cat#G1397
Sodium chloride	Sigma-Aldrich	Cat#S9888

Potassium chloride	Sigma-Aldrich	Cat#P3911
Calcium chloride dihydrate	Sigma-Aldrich	Cat#C8106
Magnesium Chloride Hexahydrate	Sigma-Aldrich	Cat#M2670
HEPES	Sigma-Aldrich	Cat#H3375
Sodium citrate	Sigma-Aldrich	Cat#1613859
Ethanol absolute anhydrous	Carlo Erba	Cat#4145872
Triton X-100	Sigma-Aldrich	Cat#X100
Tween 20	Sigma-Aldrich	Cat#P1379
Paraformaldehyde 32%	Electron Microscopy Sciences	Cat#15714
Glutaraldehyde (25 %)	Electron Microscopy Sciences	Cat#16220
Osmium tetroxide (4 %)	Electron Microscopy Sciences	Cat#19150
Acetone	Carlo Erba	Cat#400971
Uranyl acetate	Electron Microscopy Sciences	Cat#22400
Lead nitrate	E Electron Microscopy Sciences	Cat#17900
Sodium citrate	Sigma-Aldrich	Cat#S4641
Sodium cacodylate	Electron Microscopy Sciences	Cat#12300
Agarose	Sigma-Aldrich	Cat#A9414
Propylene oxide	Sigma-Aldrich	Cat#110-205
EMBED 812	Electron Microscopy Sciences	Cat#14900
MNA	Sigma-Aldrich	Cat#45347
DDSA	Sigma-Aldrich	Cat#45346
DMP30	Electron Microscopy Sciences	Cat#13600
Critical commercial assays		
RNeasy® Mini Kit Protocol	QiaGen	Cat#74104
BCA Protein Assay Kit	Thermo Fisher Scientific	Cat#23227
NuPAGE™ 4-12% Bis-Tris gels	Invitrogen	Cat# NP0321BOX
Nitrocellulose Membranes	Invitrogen	Cat#88018
LightCycler 480 SYBR Green I Master	Roche	Cat#04707516001
SuperScript III First-Strand synthesis system	Invitrogen	Cat#18080051
Protease and phosphatase inhibitors	Thermo Fisher Scientific	Cat#78429
Cell lysis buffer	Cell Signaling Technology	Cat#9803
Deposited data		
Raw and analyzed data	This paper	GEO: GSE289418
Experimental models: Cell lines		
hiPSC lines from patients carrying *LMNA* p.H222P mutation	Kindly provided by Pr. Eschenhagen, Univ ersity of Hambourg	N/A
hiPSC lines from patients carrying corrected *LMNA* p.H222P mutation (corr.H222P)	Kindly provided by Pr. Eschenhagen, Univ ersity of Hambourg	N/A
Experimental models: Organisms/strains		
Mouse: 129S2/svPasCrl wild-type	Janvier Labs	https://www.janvier-labs.com

Mouse: Mouse 129S2/svPasCrl *Lmna*$^{H222P/H222P}$	Kindly provided by Dr. Bonne[60], INSERM UMR-S 974, Paris, France	N/A
**Oligonucleotides**		
Primers for RT-qPCR, see Table S1	This paper	N/A
**Software and algorithms**		
ImageJ/Fiji	Schindelin et al.[57]	https://fiji.sc
Ionoptix Software – IonWizard 7.3	IonOptix	https://www.ionoptix.com/products/software/ionwizard-core-and-analysis/
Zen Software	Zeiss	https://www.zeiss.com/microscopy/fr/produits/logiciel/zeiss-zen.html
MorphoScript	Homan et al.[56]	https://github.com/heleneayari/Morphoscript
MetaMorph 7.10	Molecular Device	https://support.moleculardevices.com/s/article/MetaMorph-Microsoft-Windows-Operating-System-Compatibility
CellPose v2.2.2	Stringer et al.[58]	https://github.com/MouseLand/cellpose
QuPath v0.3.2	Bankhead et al.[59]	https://qupath.github.io
Fastp v0.20.0	Chen et al.[62]	https://github.com/OpenGene/fastp
Star v2.7.5a	Dobin et al.[63]	https://github.com/alexdobin/STAR
Samtools v1.13	Li et al.[64]	http://samtools.sourceforge.net/
Qualimap v2.2.2b	García-Alcade et al.[65]	https://github.com/refinery-platform/qualimap2
FeatureCounts v2.0.1	Liao et al.[66]	https://subread.sourceforge.net/featureCounts.html
Mutliqc v1.13	Ewels et al.[67]	https://github.com/MultiQC/MultiQC
DESeq2 package	Love et al.[68]	https://bioconductor.org/packages/release/bioc/html/DESeq2.html
Enrichr	Chen et al.[69], Kuleshov et al.[70], Xie et al.[71]	https://maayanlab.cloud/Enrichr/
STRING v12.0	Szklarczyk et al.[72]	https://string-db.org
ggplot2 package	Wickham[73]	https://ggplot2.tidyverse.org
RStudio		https://github.com/rstudio/rstudio
Prism 10	GraphPad	https://www.graphpad.com/features

Image Lab	Bio-Rad	https://www.bio-rad.com/fr-fr/product/image-lab-software?ID=KRE6P5E8Z
Iox2 Software	Emka Technologies	https://www.emkatech.com/product/iox2-software/
ecgAUTO	Emka Technologies	https://www.emkatech.com/product/ecgauto-software/
DigitalMicrograph	GATAN	
Other		
Microscopy dishes	Ibidi	Cat#81156
PD6 Culture Dishes	Dutscher	***Cat#067003***
TPP - 6-well plates (1x42)	Dutscher	***Cat#992106***
Zeiss LSM900	Zeiss	https://www.zeiss.com/microscopy/fr/produits/microscopes-optiques/microscopes-confocaux/lsm-900-pour-les-materiaux.html
Nikon Ti2	Nikon	https://www.microscope.healthcare.nikon.com/fr_EU/products/inverted-microscopes/eclipse-ti2-series
Prime 95B Scientific CMsOS (sCMsOS) camera	Teledyne Vision Solutions	https://www.teledynevisionsolutions.com/products/prime-95b/?vertical=tvs-photometrics&segment=tvs
Live super-resolution module (Live-SR 3D)	Gataca Systems	https://www.gataca-systems.com/products/gataca-products/super-resolution-module/
Vivid 7 Dimension/Vivid7 PRO ultrasound	GE HealthCare	
ecgTUNNEL	Emka Technologies	https://www.emkatech.com/product/ecgtunnel/

Bioanalyzer 2100	Agilent	https://www.agilent.com/en/product/automated-electrophoresis/bioanalyzer-systems/bioanalyzer-instrument/2100-bioanalyzer-instrument-228250
NovaSeq6000	Illumina	https://www.illumina.com/systems/sequencing-platforms/novaseq.html
ChemiDoc MP Imaging System	Bio-Rad	https://www.bio-rad.com/fr-fr/product/chemidoc-mp-imaging-system?ID=NINJ8ZE8Z
EVOS™ M5000 microscope	Invitrogen	Cat#AMF5000
Transmission electron microscope JEM-1011	JEOL	
Erlangshen 1000 digital camera	GATAN	
ultramicrotome UC7	Leica	
JEM-1400Flash	JEOL	
EM-SIS – high-resolution digital camera	Xarosa	
IonOptix acquisition system FSI700 fluorescence with PMT400	IonOptix	

SUPPLEMENTAL FIGURES

Figure S1 | Generation of *LMNA* H222P isogenic hiPSC control (*LMNA* corr.H222P).

(A) Flow cytometry analyzing the cell pluripotency using the SSEA-3 marker. **(B)** Validation of the genetic correction of the *LMNA* p.H222P mutation by CRISPR/Cas9 and checking of the off-targets. **(C)** The isogenic control *LMNA* corr.H222P did not show any genetic instability after the *LMNA* p.H222P mutation correction by CRISPR/Cas9.

Figure S2 | Mutated A-type lamins exhibited altered calcium handling.

Traces representing calcium handling measurement, translating a higher diastolic calcium in *LMNA* H222P compared to *LMNA* corr.H222P hiPSC-CMs.

Figure S3 | Mutated A-type lamins exhibited altered contractile function.

Representative contraction traces of *LMNA* H222P and *LMNA* corr.H222P hiPSC-CMs. Overlaid representative contraction amplitude traces recorded from hiPSC-CMs show the isogenic control (grey) and *LMNA* H222P mutant (blue) lines. Traces represent the median displacement over time obtained by video-edge capture. The control cells exhibit larger contraction amplitudes and faster relaxation kinetics compared with the LMNA mutant, which displays reduced amplitude and slower relaxation. Time is expressed in milliseconds (ms), and contraction amplitude is shown in arbitrary units (A.U.).

Figure S4 | Mutated A-type lamins did not alter the chromosomes 9, 11, 16, 17, and 18 spatial positioning.

Top: Representative images of chromosome painting experiments. Scale bar 5 μm. **Bottom:** Heatmaps and graphs representing the results from the chromosome painting experiment. Source data are provided as a Source Data file. Chromosome painting experiment: n_{H222P}= 2, $n_{corr.H222P}$= 2

Figure S5 | Common dysregulation of extracellular matrix gene expression in both *LMNA* H222P hiPSC-CMs and *Lmna*$^{H222P/H222P}$ mice

(A) Principal component analysis (PCA) of mice RNA-seq samples. **(B)** Volcano plot representing DEGs between *Lmna*$^{H222P/H222P}$ and WT mice. The dotted lines correspond to the threshold used for the analysis. **(C)** Graphical representation of the first 20 biological processes associated with DEGs in mice. Generated with STRING v12.0. (**D)** Venn diagram and table recapitulating the common biological processes associated with DEGs in common between hiPSC-CMs and mice. Graphical representation of ECM-related DEGs per chromosome.

Source data are accessible in GSE289418. RNA-seq experiment: n_{H222P}= 2, $n_{corr.H222P}$= 3, n_{Lmna}= 3, n_{WT}= 2; threshold, padj 0.05, log2FC 0.5.

Figure S6 | Overexpression of Loxl2 in cardiac cells expressing *LMNA* mutations.

Gene expression of *Loxl2* from publicly available RNAseq dataset from iPSCs, mouse model, and human cardiac tissue expressing mutated A-type lamins (mean ± SEM). Illustrations generated by BioRender.

Figure S1

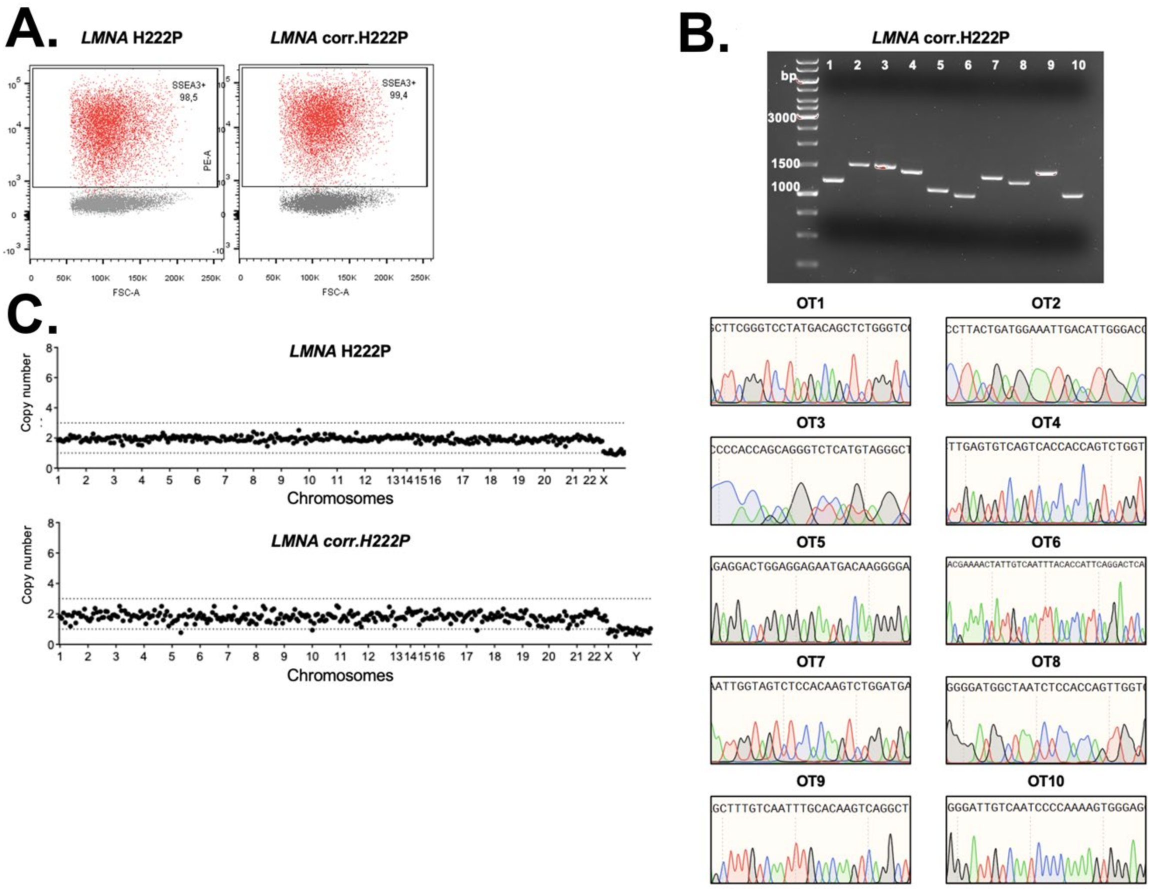
A.
LMNA H222P
LMNA corr.H222P
SSEA3+
98,5
SSEA3+
99,4
PE-A
FSC-A
B.
LMNA corr.H222P
bp
3000
1500
1000
1 2 3 4 5 6 7 8 9 10
OT1
OT2
OT3
OT4
OT5
OT6
OT7
OT8
OT9
OT10
C.
LMNA H222P
LMNA corr.H222P
Copy number
Chromosomes

Figure S2

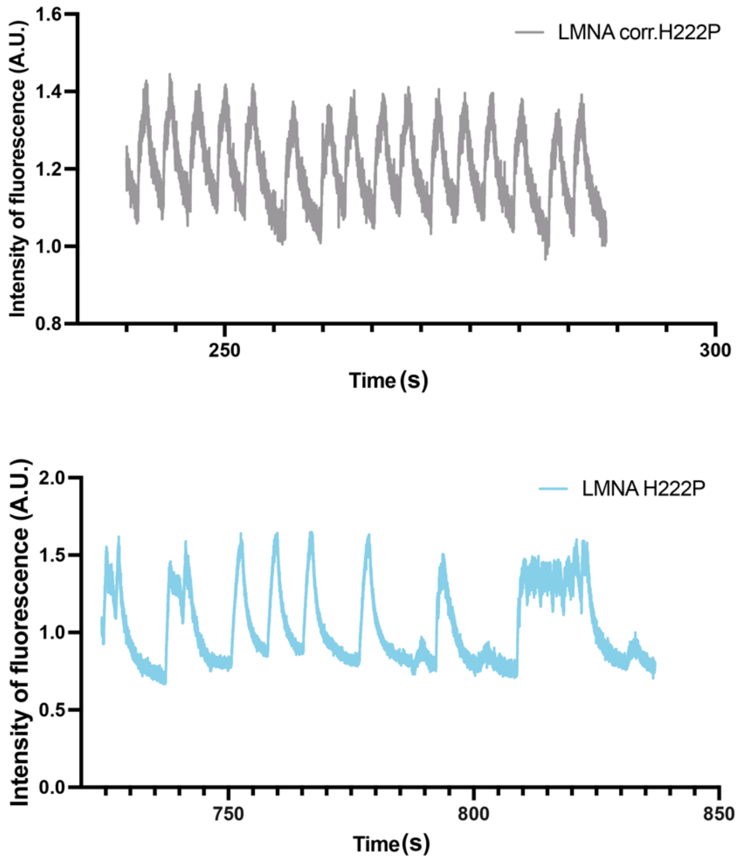
LMNA corr.H222P
Intensity of fluorescence (A.U.)
1.6
1.4
1.2
1.0
0.8
250
300
Time (s)
LMNA H222P
Intensity of fluorescence (A.U.)
2.0
1.5
1.0
0.5
0.0
750
800
850
Time (s)

Figure S3

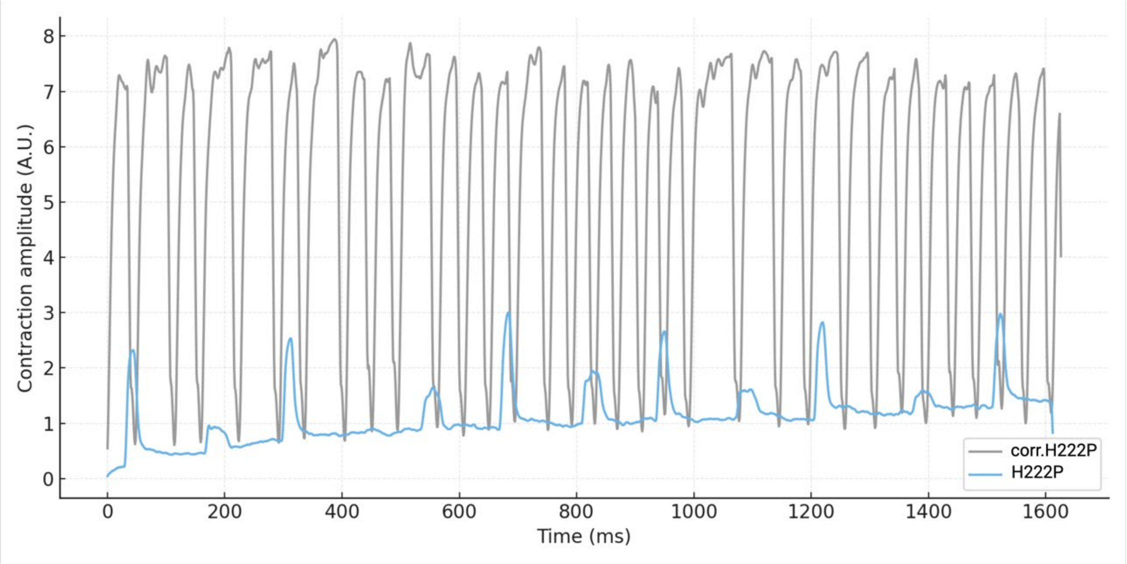

Contraction amplitude (A.U.)
Time (ms)
corr.H222P
H222P
0
1
2
3
4
5
6
7
8
0
200
400
600
800
1000
1200
1400
1600

Figure S4

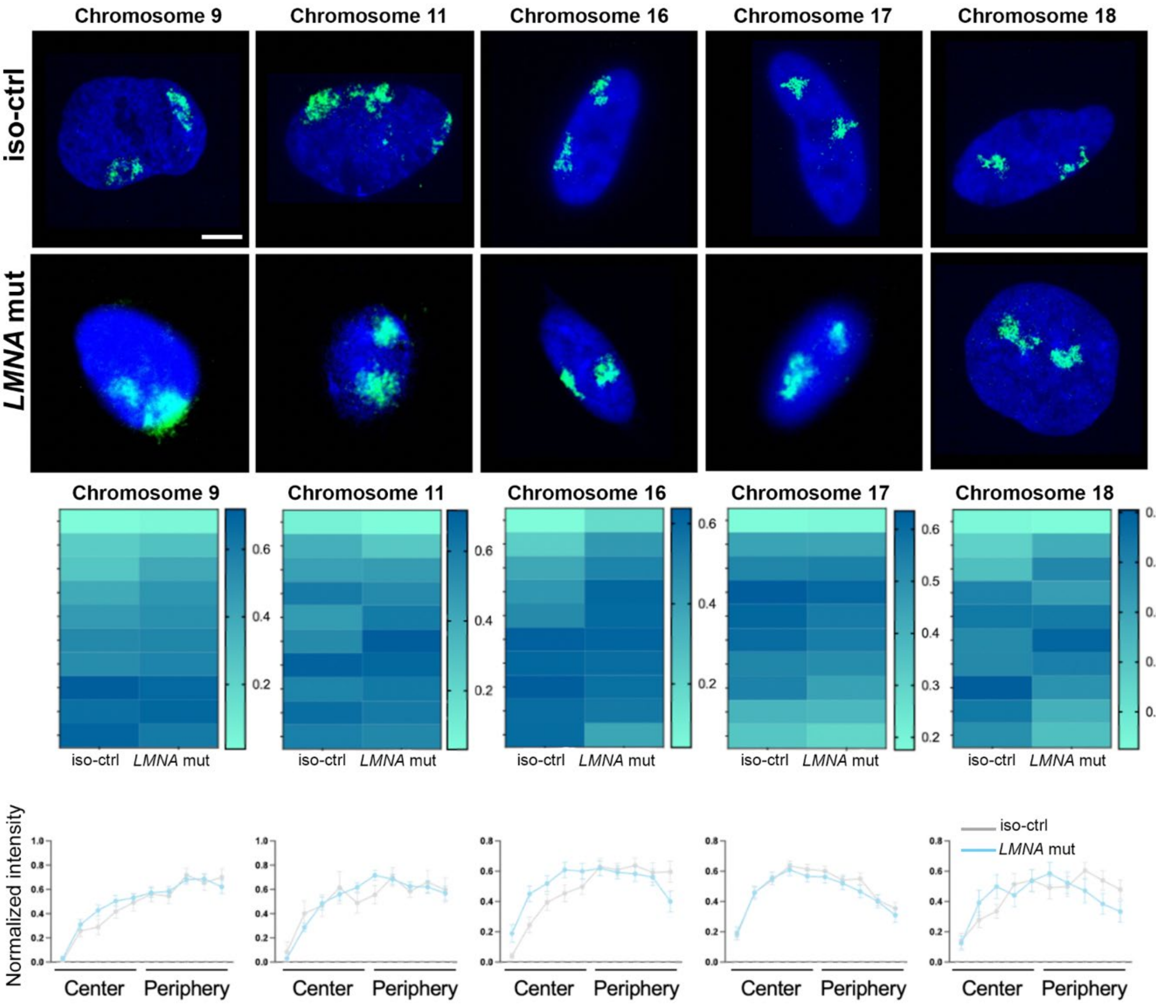

Chromosome 9
Chromosome 11
Chromosome 16
Chromosome 17
Chromosome 18
iso-ctrl
LMNA mut
Chromosome 9
Chromosome 11
Chromosome 16
Chromosome 17
Chromosome 18
iso-ctrl
LMNA mut
Normalized intensity
Center
Periphery
iso-ctrl
LMNA mut

Figure S5

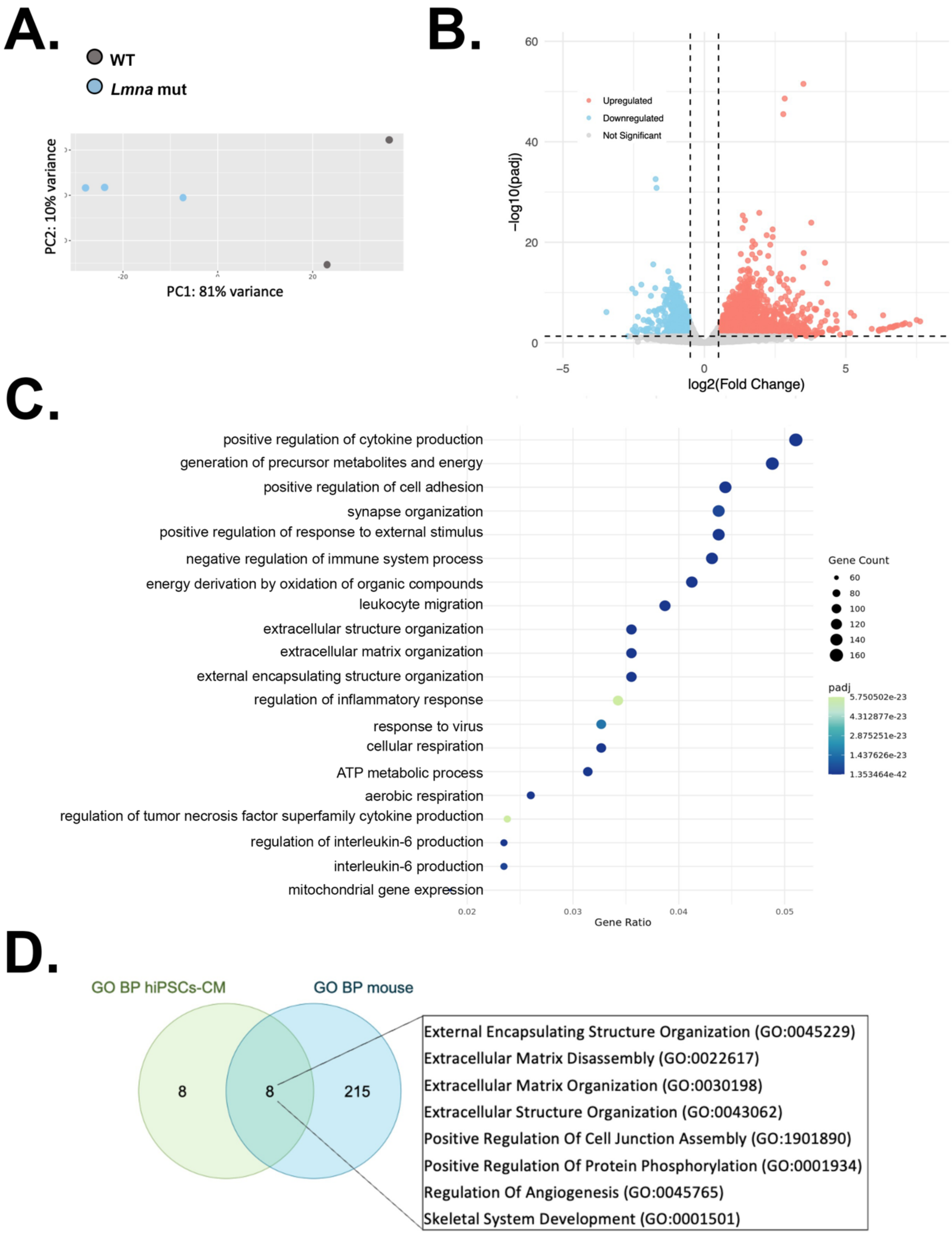
A.
WT
Lmna mut
PC2: 10% variance
PC1: 81% variance
-20
20
B.
Upregulated
Downregulated
Not Significant
−log10(padj)
log2(Fold Change)
60
40
20
0
−5
0
5
C.
positive regulation of cytokine production
generation of precursor metabolites and energy
positive regulation of cell adhesion
synapse organization
positive regulation of response to external stimulus
negative regulation of immune system process
energy derivation by oxidation of organic compounds
leukocyte migration
extracellular structure organization
extracellular matrix organization
external encapsulating structure organization
regulation of inflammatory response
response to virus
cellular respiration
ATP metabolic process
aerobic respiration
regulation of tumor necrosis factor superfamily cytokine production
regulation of interleukin-6 production
interleukin-6 production
mitochondrial gene expression
0.02
0.03
0.04
0.05
Gene Ratio
Gene Count
60
80
100
120
140
160
padj
5.750502e-23
4.312877e-23
2.875251e-23
1.437626e-23
1.353464e-42
D.
GO BP hiPSCs-CM
GO BP mouse
8
8
215
External Encapsulating Structure Organization (GO:0045229)
Extracellular Matrix Disassembly (GO:0022617)
Extracellular Matrix Organization (GO:0030198)
Extracellular Structure Organization (GO:0043062)
Positive Regulation Of Cell Junction Assembly (GO:1901890)
Positive Regulation Of Protein Phosphorylation (GO:0001934)
Regulation Of Angiogenesis (GO:0045765)
Skeletal System Development (GO:0001501)

Figure S6

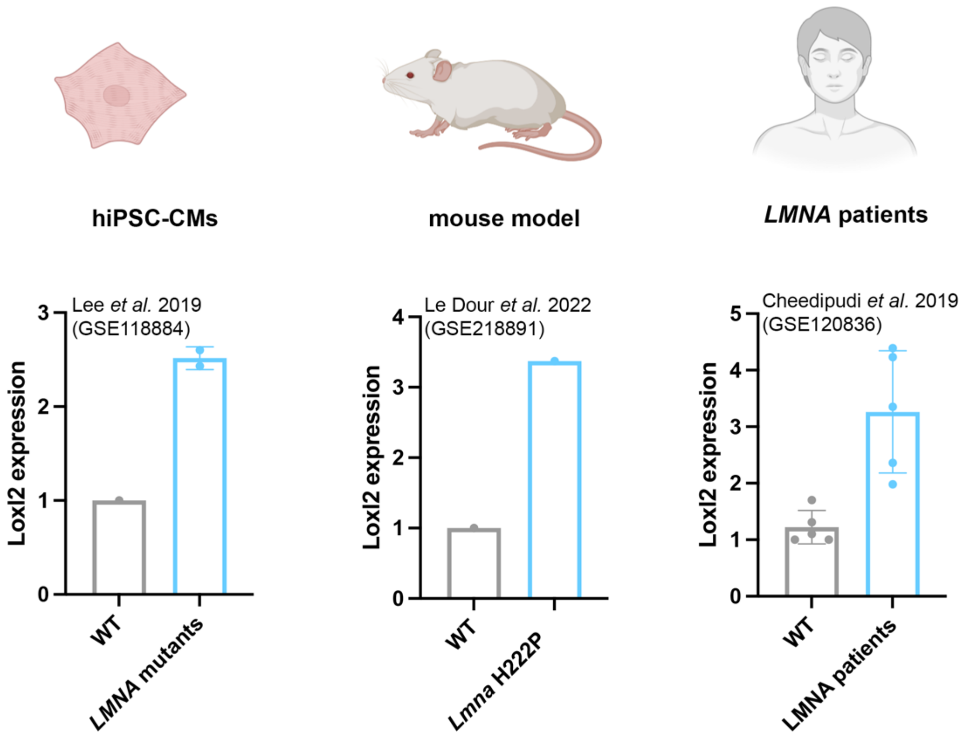

hiPSC-CMs
mouse model
LMNA patients
Lee et al. 2019 (GSE118884)
Le Dour et al. 2022 (GSE218891)
Cheedipudi et al. 2019 (GSE120836)
Loxl2 expression
WT
LMNA mutants
Lmna H222P
LMNA patients
0
1
2
3
4
5

SUPPLEMENTAL TABLES

Table S1 | RT-qPCR primers sequences

	forward (5'-3')	reverse (5'-3')
MyH7	5'-gggtcatccaatattttgctgt-3'	5'-ggttggcttggatgatttga-3'
NppA	5'-gcttccaggccatattggag-3'	5'-gggggcatgacctcatctt-3'
Tnni1	5'-gctgagaaggtgcgttacctc-3'	5'-agctctcggcacaagtcct-3'
Col1a2	5'-ccgtgcttctcagaacatca-3'	5'-,gagcagccatcgactaggac-3'
Col3a1	5'-tgtggacattggccctgttt-3'	5'-tggtcacttgcactggttga-3'
Sdc-1	5'-ttcagccaaggttttacagg-3'	5'-caagatgtgctagagtcccaa-3'
Rplpo	5'-ctccaagcagatgcagcaga-3'	5'-atagccttgcgcatcatggt-3'

UNCROPPED IMMUNOBLOTS

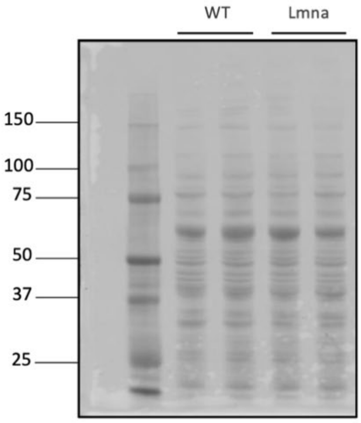

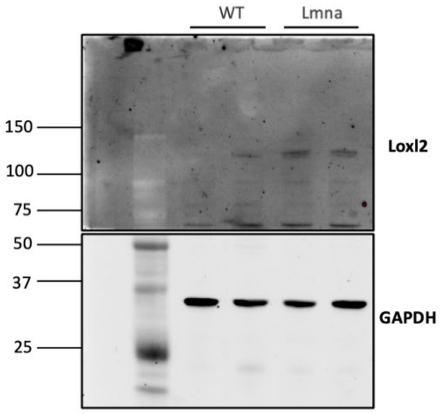

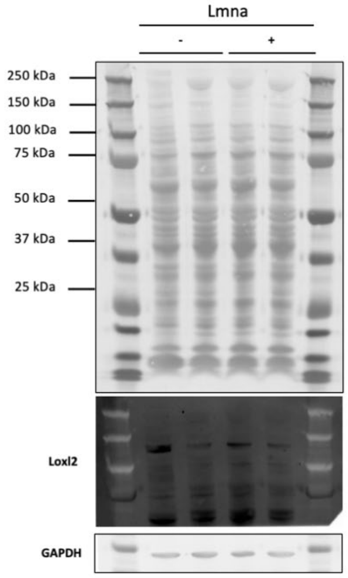

Supplement: Supplementary file 2 [file hhf-19-e013806-s003.pdf]
